# Real-World Effectiveness of Nirsevimab Against RSV-LRTI and Bronchiolitis Hospitalization: Results from the 2024–2025 Italian Universal Immunization Campaign in Newborns

**DOI:** 10.3390/v18090982

**Published:** 2026-09-07

**Authors:** Cinzia Auriti, Camilla Gizzi, Matteo Riccò, Roberto Bellù, Mario Giuffrè, Adele Fabiano, Caterina Cacace, Paola Cavicchioli, Armando Cuttano, Susanna Di Valerio, Cristiana Germini, Marcello Napolitano, Giulia Paviotti, Chiara Peila, Serafina Perrone, Simona Pesce, Gianfranco Scarpelli, Grazia Scutti, Giuseppina Spanedda, Alex Staffler, Massimo Agosti, on behalf of the Italian Working Group on RSV Prophylaxis of Italian Society of Neonatology

**Affiliations:** 1 Faculty of Medicine, General and Specialist Pediatrics, Saint Camillus International University of Health and Medical Sciences (Unicamillus), 00131 Rome, Italy; cinzia.auriti@unicamillus.org; 2 Neonatal Intensive Care Unit S., Eugenio Hospital, 00144 Rome, Italy; 3 Servizio di Prevenzione e Sicurezza Negli Ambienti di Lavoro (SPSAL), AUSL-IRCCS di Reggio Emilia, 42122 Reggio Emilia, Italy; 4 Neonatal Intensive Care and Neonatal Units, Lecco Hospital of Lecco ASST, 23900 Lecco, Italy; r.bellu@asst-lecco.it; 5 Neonatal Intensive Care Unit and Pediatrics, G. D’Alessandro Department, Palermo University, 90127 Palermo, Italy; mario.giuffre@unipa.it; 6 Neonatal Intensive Care Unit, San Giovanni Hospital, 00184 Rome, Italy; adyfab@gmail.com; 7 Pediatric Unit and NICU, “Barone Romeo” Hospital of ASP Messina, 98123 Messina, Italy; cacacecaterina@gmail.com; 8 Pediatric Unit, dell’Angelo Hospital Mestre, 30174 Venezia, Italy; paola.cavicchioli@aulss3.veneto.it; 9 Neonatal Intensive Care Unit, University Hospital Pisa, 56100 Pisa, Italy; cuttano@unipi.it; 10 Neonatal Intensive Care Unit, Santo Spirito Hospital, 65124 Pescara, Italy; susanna.divalerio@asl.pe.it; 11 Neonatal Unit, Perugia University Hospital, 06156 Perugia, Italy; cristiana.germini@ospedale.perugia.it; 12 Neonatal Intensive Care and Neonatal Units, Evangelico Betania Hospital, 80100 Napoli, Italy; mnapolitano64@gmail.com; 13 Neonatal Intensive Care Unit, University Hospital of Udine, 33100 Udine, Italy; giulia.paviotti@asufc.sanita.fvg.it; 14 Neonal Unit, Department of Public Health and Pediatric Sciences, Turin University, 10126 Turin, Italy; chiara.peila@unito.it; 15 Neonatology Unit, Pietro Barilla Children’s Hospital, University of Parma, 43125 Parma, Italy; serafina.perrone@unipr.it; 16 Neonatal Intensive Care Unit, San Carlo Hospital, 85100 Potenza, Italy; pesce.simona@me.com; 17 Neonatal Intensive Care Unit and Pediatrics, Cosenza Hospital, 87100 Cosenza, Italy; g.scarpelli@aocs.it; 18 Neonatal Intensive Care Unit, “Salesi” Hospital, 60126 Ancona, Italy; grazia.scutti@libero.it; 19 Neonatal Intensive Care Unit, Sassari University Hospital, 07100 Sassari, Italy; giuseppina.spanedda@aouss.it; 20 Neonatal Intensive Care Unit, Division of Neonatology, Hospital of Bolzano, Teaching Hospital of Paracelsus Medical University, 39100 Bolzano, Italy; alex.staffler@asbz.it; 21 Neonatal Intensive Care and Neonatal Units, Hospital of Varese—University of Insubria, 21100 Varese, Italy; massimoagostino.agosti@gmail.com

**Keywords:** RSV, nirsevimab, lower respiratory tract infection, bronchiolitis, newborn, infant, hospitalization, respiratory support, prophylaxis

## Abstract

Respiratory syncytial virus (RSV) remains a leading cause of lower respiratory tract infection (LRTI) and hospitalization in early infancy. Following the introduction of universal nirsevimab prophylaxis in Italy during the 2024–2025 RSV season, we evaluated its real-world effectiveness against RSV-associated hospitalization and severe clinical outcomes within a test-negative design framework. We conducted a multicenter retrospective test-negative study across 88 Italian neonatal centers, including infants hospitalized for LRTI from 1 September 2024 to 30 April 2025. Clinical data were collected from hospital records using a standardized electronic questionnaire. RSV-positive hospitalized infants were considered cases, and RSV-negative hospitalized infants served as controls. Secondary outcomes included respiratory support and Neonatal Intensive Care Unit (NICU) admission. Effectiveness was estimated using multivariable logistic regression and targeted maximum likelihood estimation. Overall, 737 infants were included, of whom 306 (41.52%) had previously received nirsevimab. RSV infection was identified in 398 infants (54.00%), including 104/306 (33.99%) nirsevimab-immunized and 294/431 (68.21%) non-immunized infants (*p* < 0.001). Immunized infants also required respiratory support less frequently (55.23% vs. 70.07%; *p* < 0.001), with lower use of high-flow oxygen therapy (40.85% vs. 48.96%; *p* = 0.036) and Continuous Positive Airway Pressure (CPAP) (18.95% vs. 33.87%; *p* < 0.001). Within the test-negative design framework, adjusted effectiveness against RSV-associated hospitalization was estimated at 78.3%. Among hospitalized infants, previous nirsevimab immunization was additionally associated with adjusted relative reductions in the odds of respiratory support and NICU admission of 80.2% and 64.3%, respectively. In conclusion, among infants hospitalized for LRTI in routine Italian clinical practice, previous nirsevimab immunization was associated with lower odds of RSV positivity and a reduced need for respiratory support. Although these findings provide further evidence of the real-world effectiveness of nirsevimab against severe RSV disease, further studies are needed to provide direct estimates of hospitalization incidence in immunized and non-immunized source populations.

## 1. Introduction

Respiratory syncytial virus (RSV; genus orthopneumovirus) is an enveloped, pleomorphic virus (average size: 150 nm; range: 120–300 nm) that usually targets the respiratory tract [1]. Due to its filamentous, single-stranded, negative-sense RNA genome of 15–16 kb [2,3,4,5], RSV is classified within the order *Mononegavirales* (which also includes significant human pathogens belonging to the families Paramyxoviridae, Rhabdoviridae, Filoviridae), and more specifically within the family Pneumoviridae [6] alongside human Metapneumovirus (hMPV). RSV and hMPV share similarities in their genome [7,8,9], viral structures and life cycles, as well as in their strategies for host interaction and immune evasion [7,9,10,11]. Since its original isolation in 1957 [12,13], RSV has been considered one of the leading causes of respiratory syndromes, including acute respiratory infections (ARI), lower respiratory tract infections (LRTI, i.e., either acute infection or illness involving the lower respiratory tract, or exacerbations of pre-existing conditions) [14], bronchiolitis (i.e., a viral LRTI characterized by acute inflammation, edema, and necrosis of epithelial cells lining small airways, and increased mucus production) [15], and hospitalizations in infants, particularly during the first months of life [16,17,18].

In this regard, before the implementation of universal preventive campaigns by means of monoclonal antibodies (mAbs) and maternal vaccines, the annual global burden of RSV was substantial. According to available figures, RSV infections led to around 33 million cases of ARI and LRTI in children aged 5 years or younger every year [3,4,19], with high hospitalization rates [20,21,22,23,24] and a substantial burden of pediatric morbidity, especially among children younger than 3 months [25].

Due to the very high occurrence of incident cases, RSV infections place considerable pressure on neonatal and pediatric healthcare services [2], with a need for supportive care, including respiratory support with continuous positive airway pressure (CPAP) devices, and even mechanical ventilation and/or extra-corporeal membrane oxygenation (ECMO). While no specific antiviral therapy is currently available for respiratory syndromes associated with RSV infections [26,27], three mAbs (i.e., palivizumab, nirsevimab, and clesrovimab) and one maternal vaccine (RSVpreF, commercial name: Abrysvo^TM^, Pfizer Europe MA EEIG, Brussels, Belgium) have been made available to prevent RSV infections and/or reduce the occurrence and the severity of complications in children and newborns [28,29]. Therefore, prevention has become a central strategy for reducing RSV-related disease burden [30].

Palivizumab was the first mAb to be licensed and used in real-world settings as a preventive option for RSV. It targets site II of the F protein and, despite its documented effectiveness, was ultimately used only among high-risk pediatric patients [31,32,33,34] because of its substantial direct and indirect costs [34,35], resulting from its short-acting design and the need for monthly, weight-dependent doses (i.e., 15 mg/kg) [32,36,37].

Universal immunization campaigns became available after the licensing of nirsevimab (Beyfortus, AstraZeneca [Södertälje, Sweden], and Sanofi [Gentilly, France]), a long-acting mAb directed against site ø within the prefusion F protein [7,38,39]. By design, nirsevimab was developed to provide passive immunization throughout a single epidemic season with one administration [40]. Since its initial commercialization, the effectiveness of nirsevimab in preventing severe LRTI [41,42,43] has been documented by several real-world studies [41,42,43,44,45,46,47,48], with an early estimate for immunization effectiveness of around 90.5% (95% CI 87.1 to 93.0) and with very limited occurrence of adverse reactions and side effects [49]. Virtually all observational studies published from 2024 onwards have confirmed these preliminary results [29], emphasizing the effectiveness of nirsevimab in reducing not only the proportion of RSV-associated hospitalizations but also overall healthcare utilization among targeted children. Following the early positive results from universal immunization campaigns, during the 2024–2025 RSV season, Italy introduced a nationwide prevention campaign offering nirsevimab to newborns and young infants, thus creating an opportunity to evaluate its effectiveness in real-world settings [50]. Since then, several Italian studies providing early estimates of nirsevimab effectiveness in real-world settings have been published [51,52,53,54,55,56,57]. Still, given the heterogeneous and highly regionalized organization of the Italian National Health Service, multicenter studies providing comprehensive nationwide coverage remain necessary to better characterize the real-world effectiveness of this preventive strategy, while also assessing its potential impact on healthcare resource utilization. The present multicenter, multiregional study was therefore designed to assess the impact of nirsevimab prophylaxis on RSV-associated hospitalizations and disease severity among infants admitted with LRTI to several Italian neonatal units during the 2024–2025 epidemic season.

## 2. Materials and Methods

### 2.1. Study Design and Population

This multicenter retrospective observational study was conducted across 88 neonatal centers in Italy using a test-negative design. The study was reported in accordance with the Strengthening the Reporting of Observational Studies in Epidemiology (STROBE) statement, and the completed STROBE checklist is provided in the Supplementary Materials (Supplementary File S1). Participating centers are detailed in Appendix A Table A1, alongside the regional timing of nirsevimab implementation during the 2024–2025 RSV season in Italy. Participating centers were neonatal units, neonatal care units, or NICUs; therefore, pediatric wards potentially admitting older infants with LRTI within the same institutions were not systematically included in the present report from the surveillance network.

According to estimates from the Italian National Institute of Statistics (ISTAT; https://demo.istat.it, accessed on 25 April 2026), during the 2024–2025 winter season, approximately 731,314 children aged <2 years and 358,173 children aged <1 year were living in Italy (Table 1). As hospital admissions in Italy are not strictly linked to patients’ place of residence, the catchment population potentially served by the participating centers cannot be precisely defined. Nevertheless, using the population of the province in which each participating center was located as a reference, the approximate potential catchment population was estimated at 509,244 children aged <2 years, corresponding to 69.63% of the national population in this age group, with a similar proportion for children aged <1 year (69.79%). These figures should be interpreted as approximate estimates of the population residing in the geographic areas potentially served by the participating centers and not as measures of actual population coverage or national healthcare coverage.

Standardized clinical and demographic data on newborns and infants hospitalized because of LRTI were retrieved by means of a structured electronic questionnaire (Appendix A Table A2) completed by participating centers based on hospital discharge records.

The inclusion criteria were as follows:

(1) Hospital admission due to LRTI between 1 September 2024 and 30 April 2025, i.e., the first epidemic season in which nirsevimab was introduced as universal prophylaxis in Italy;
(2) Availability of demographic data including date of birth; Italian region of origin (categorized as: Northern Italy, Central Italy, Southern Italy, Main Islands);
(3) Age ≤ 24 months at the time of admission;
(4) Availability of immunization status by means of nirsevimab (i.e., immunized vs. not immunized) and/or palivizumab as retrieved by the database of the relevant regional health authority or, where unavailable, from documentation provided by the parents;
(5) Availability of clinical data including month of diagnosis; comorbidities, duration of hospitalization, respiratory support requirements, and neonatal intensive care unit (NICU) admission;
(6) Having been tested for RSV status by means of nasopharyngeal swab using a real-time quantitative polymerase chain reaction (RT-PCR) or antigenic testing.

The exclusion criteria were: (1) lack of any of clinical and/or demographic data; (2) age > 24 months at the time of hospital admission; (3) having been immunized with at least one dose of palivizumab; (4) documented maternal vaccination with the RSVpreF vaccine; (5) immunization status reported only verbally by parents or caregivers; (6) the lack of virological diagnostic test.

All eligible admissions meeting the predefined inclusion criteria during the study period were consecutively included by each participating center. A preliminary data-quality check was performed to identify potentially duplicated records, including those related to inter-hospital transfers. In such cases, for the aims of the present analysis, only the record provided by the discharging facility was eventually retained.

Given the persistent lack of a standardized and widely accepted definition of LRTI [7,14], particularly in pediatric age groups [49,56], the following working definition was implemented: any child hospitalized because of bronchiolitis, bronchitis, pneumonia, or bronchopneumonia was considered to have LRTI. Infants admitted due to noninfectious respiratory presentations (e.g., choking, lung malformations, wheezing, or allergic bronchospasm attacks) were conversely not included. For descriptive purposes, breakthrough RSV infection was operationally defined as laboratory-confirmed RSV infection occurring in an infant with a documented history of previous nirsevimab administration. As the exact date of nirsevimab administration was not available, this definition did not require a predefined interval between prophylaxis and RSV infection and should not be interpreted as evidence of prophylaxis failure after an adequate post-administration protection interval.

### 2.2. Exposure and Outcomes

The primary directly observed outcome was RSV positivity among infants hospitalized for LRTI. Infants with laboratory-confirmed RSV infection were considered cases, whereas infants hospitalized for LRTI who tested negative for RSV served as controls. According to the test-negative design, this comparison was used to assess the association between prior nirsevimab prophylaxis and RSV positivity among infants hospitalized for LRTI.

Secondary outcomes included length of hospital stay, need for CPAP, invasive respiratory support, NICU admission, and mortality. Secondary outcomes were assessed according to nirsevimab immunization status.

### 2.3. Statistical Analysis

As a preliminary step, a descriptive analysis was performed, providing data for the whole sample, by RSV status (positive vs. negative), and by nirsevimab immunization status (yes vs. no).

Continuous variables were reported as mean ± standard deviation (SD) and were preliminarily checked for normal distribution by means of the K2 test. A significance level of α = 0.10 was implemented to increase the test’s sensitivity to departures from normality. In other words, variables with a K2 test *p*-value of <0.100 were defined as not normally distributed, leading to their analysis using the Mann–Whitney or Kruskal–Wallis tests, while correlation analysis was performed using Spearman’s rank test. Conversely, variables associated with a K2 test *p*-value of ≥0.100 were considered normally distributed, and their correlation was assessed using Pearson’s correlation coefficient. Levene’s test was then used to assess the equality of variances (i.e., homoscedasticity) of the data. Data exhibiting homoscedasticity and normality were compared between groups using an independent-samples *t*-test. Heteroscedastic normally distributed data were compared between groups using Welch’s test (unequal-variance *t*-test). In cases of multiple comparisons, Analysis of Variance (ANOVA) with post hoc Dunnett’s test was performed.

Categorical variables were reported as counts and percentages. The distribution of categorical variables between cases and controls and according to nirsevimab immunization status was primarily assessed using the chi-squared test. Missing values for the assessed variables were handled using listwise deletion, i.e., observations with incomplete data were excluded from the specific analysis in which the missingness occurred. In all analyses, a *p* value < 0.05 was considered statistically significant. Univariate comparisons used for descriptive characterization of the study population were considered exploratory; no formal adjustment for multiple comparisons was applied, and individual marginal *p*-values were therefore not interpreted as confirmatory evidence.

Two distinct exploratory analyses were then performed. First, RSV positivity was additionally compared across age strata among nirsevimab-immunized infants to assess whether the occurrence of RSV infection after previous immunization varied according to age at hospitalization. Immunized infants aged 7–29 days were used as the reference group. This analysis was distinct from the age-stratified analysis of nirsevimab effectiveness. Second, three distinct binary logistic regression models were considered for an exploratory multivariable analysis of the factors associated with RSV-positive status (Model I), need for respiratory support (Model II), and NICU admission (Model III). Irrespective of their statistical significance in univariate analysis, age at admission, month of hospitalization, geographic region, comorbidities, respiratory support, NICU admission, and nirsevimab prophylaxis status were included a priori, as appropriate, as explanatory variables as follows:

- Model I: outcome variable: RSV-positive status; explanatory variables: age at admission, month of hospitalization, comorbidities, geographic region, previous nirsevimab administration.
- Model II: outcome variable: need for respiratory support; explanatory variables: age at admission, month of hospitalization, comorbidities, geographic region, previous nirsevimab administration.
- Model III: outcome variable: NICU admission; explanatory variables: age at admission, month of hospitalization, comorbidities, geographic region, previous nirsevimab administration.

Consistently with the conventional test-negative design framework, crude immunization effectiveness (IE) was estimated as (1 − OR) × 100, where the OR represented the odds of prior nirsevimab immunization among RSV-positive cases relative to RSV-negative controls. To account for potential confounding, adjusted IE was subsequently derived using Targeted Maximum Likelihood Estimation (TMLE). TMLE is a semiparametric estimation approach that combines outcome regression with modeling of the exposure mechanism and is doubly robust, in that consistent estimation can be retained if either nuisance model is correctly specified, under the remaining identification assumptions. In the present analyses, TMLE was performed using the *tmle* package in R, with Super Learner implemented through the *SuperLearner* package. Laboratory-confirmed RSV status was specified as the binary outcome (Y), previous nirsevimab immunization as the exposure (A), and age at admission, prematurity, month of hospitalization, and geographic region as adjustment covariates (W). Both the initial outcome regression (Q) and the exposure mechanism (g) were estimated using Super Learner, with SL.glm and SL.mean specified as candidate learners and using the default cross-validation settings. The targeting procedure estimated the marginal counterfactual probabilities of RSV positivity under nirsevimab exposure and non-exposure among infants hospitalized for LRTI included in the test-negative study population. These estimates were used to derive the corresponding marginal odds ratio and its 95% confidence interval, and adjusted IE was calculated as (1 − OR) × 100. In the present observational setting, TMLE was implemented to improve adjustment for measured baseline confounding. A parsimonious Super Learner library including SL.glm and SL.mean was used for nuisance parameter estimation. The same TMLE specification and adjustment set were additionally applied to respiratory support and NICU admission as secondary binary outcomes.

Statistical analyses were performed using IBM SPSS Statistics 27.0 for Macintosh (IBM Corp., Armonk, NY, USA), GraphPad Prism Version 10.6.1 (799) (GraphPad Software LLC, San Diego, CA, USA), R (version 4.4.1) [55], and RStudio 2026.01.0 Build 392 (Posit Software PBC, Boston, MA, USA) using the packages *fmsb* (version 0.7.6), *ggplot2* (version 4.0.1), *ggpubr* (version 0.6.2), *tmle* (version 2.1.1) and *SuperLearner* (version 2.0-40).

## 3. Results

### 3.1. Main Characteristics of Study Population

**Figure 1:**
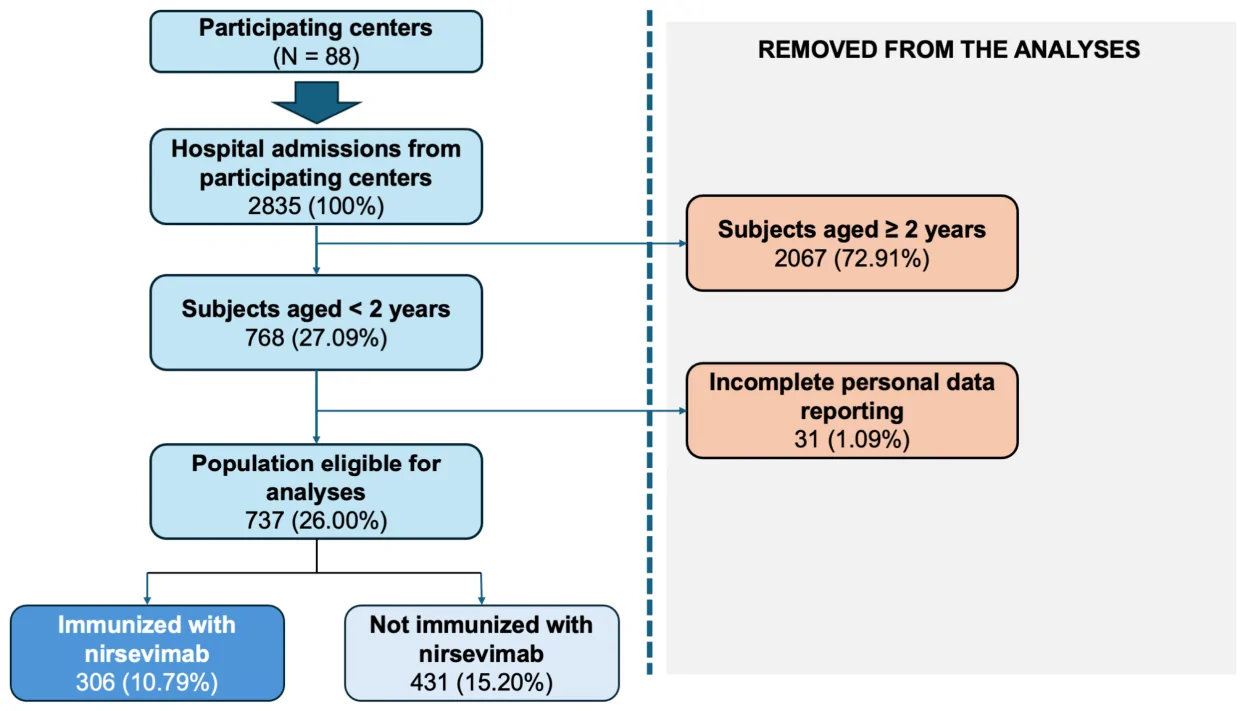
Flowchart of subjects participating in the present study.

During the assessed timeframe, participating centers reported a total of 2835 hospital admissions due to LRTI in children aged <5 years. Of these, 768 were reported in children aged 24 months or less; after inclusion and exclusion criteria were implemented, a total of 737 hospitalized newborns and infants were included in the final analysis (Figure 1). Of these, 398 (54.00%) had a documented RSV infection; 306 (41.52%) had received nirsevimab prophylaxis, whereas 431 had not (58.48%).

**Figure 2:**
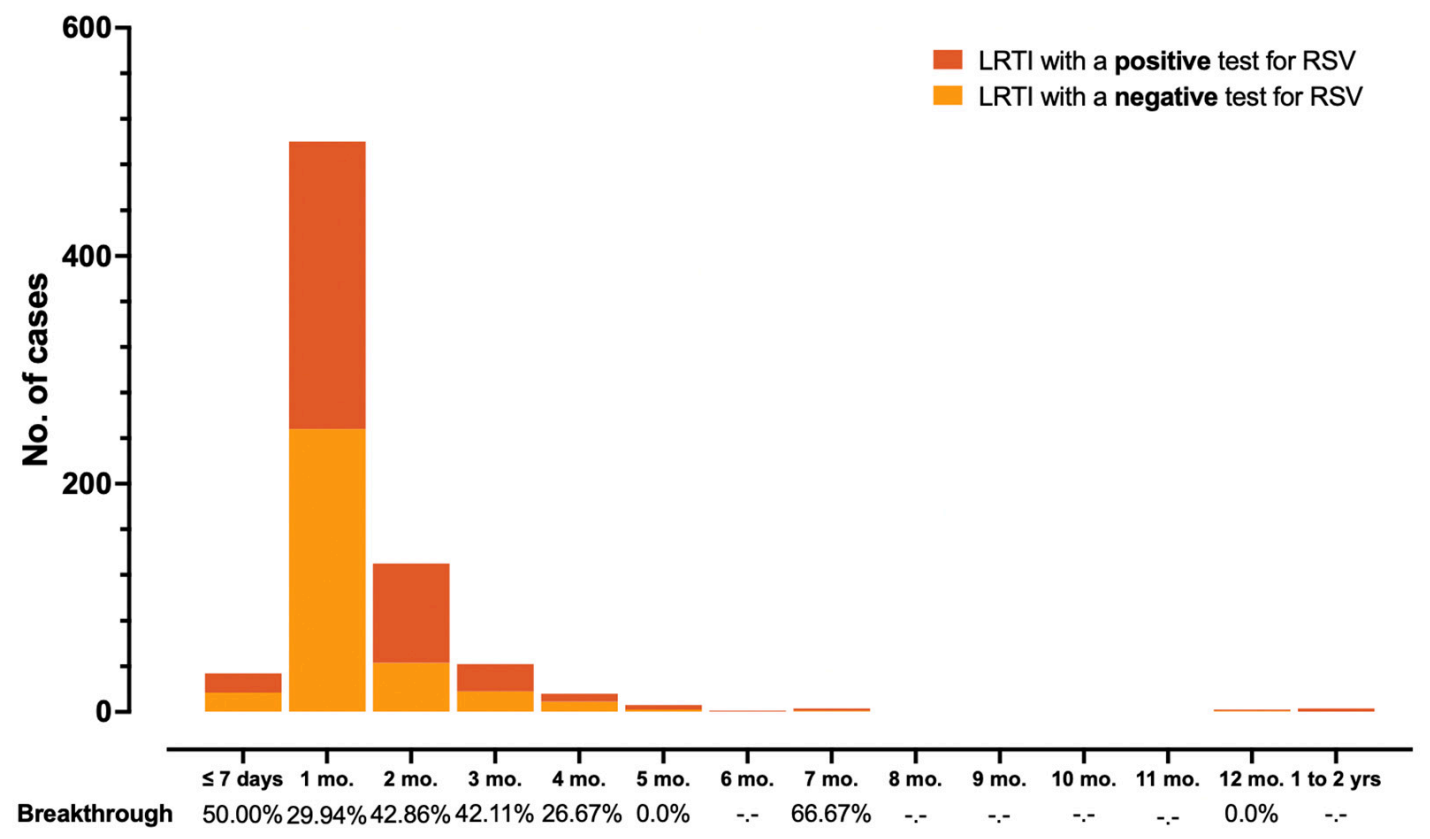
Distribution of cases of lower respiratory tract infections (LRTIS) by age at hospital admission in children immunized by means of nirsevimab. Breakthrough rate was defined for descriptive purposes as the proportion of RSV-positive cases among infants with documented previous nirsevimab administration, irrespective of the unknown interval between nirsevimab administration and hospitalization.

The mean age at admission was 31.97 ± 38.44 days (median 23.00, interquartile range [IQR] 16.50–32.50), with a markedly right-skewed distribution (K2 test 910.2, *p* < 0.001; Appendix A Figure A2). More than half of sampled LRTI cases were infants aged up to one month of age (72.46%), and most infants were younger than three months (90.09%), showing that, within the present study population, hospitalizations were strongly concentrated in early infancy (Figure 2).

The proportion of RSV cases mirrored this distribution, with most cases occurring during the first month of age (78.17%), and 96.17% of all RSV cases clustered during the first three months of age. In this regard, breakthrough rate (i.e., episodes of LRTI associated with a documented diagnosis of RSV infection despite prophylaxis with nirsevimab) was particularly high during the first week of age (50.00%), with rates of 29.95% during the first month, 42.86% during the second month, and 42.11% during the third month, decreasing to 26.67% between the fourth and the 24th month.

**Figure 3:**
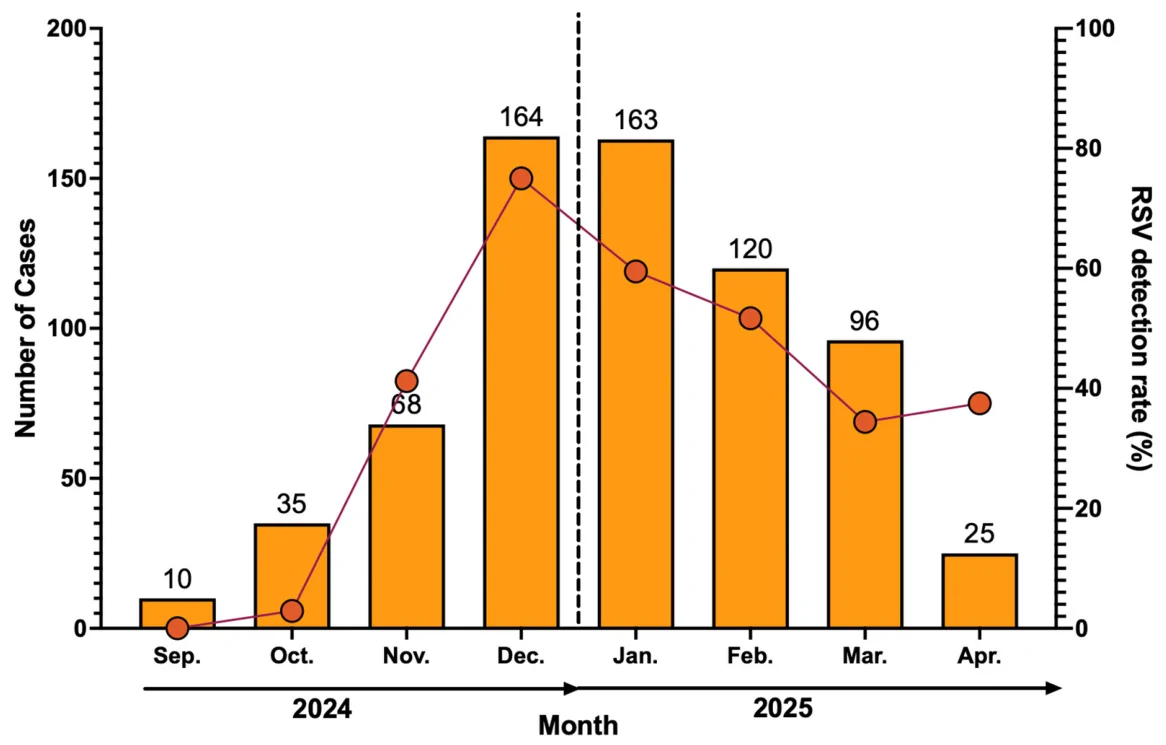
Seasonal trend of hospitalizations due to lower respiratory tract infections from the participating centers by RSV infection status (*n* = 737). Dashed line represents the separation between calendar years 2024 and 2025.

The seasonal distribution of cases showed that 80.32% of hospitalizations occurred between December and April. RSV detection rates rose sharply from September onward, peaking in December (75.69%), and subsequently declined during the following months (Figure 3).

**Figure 4:**
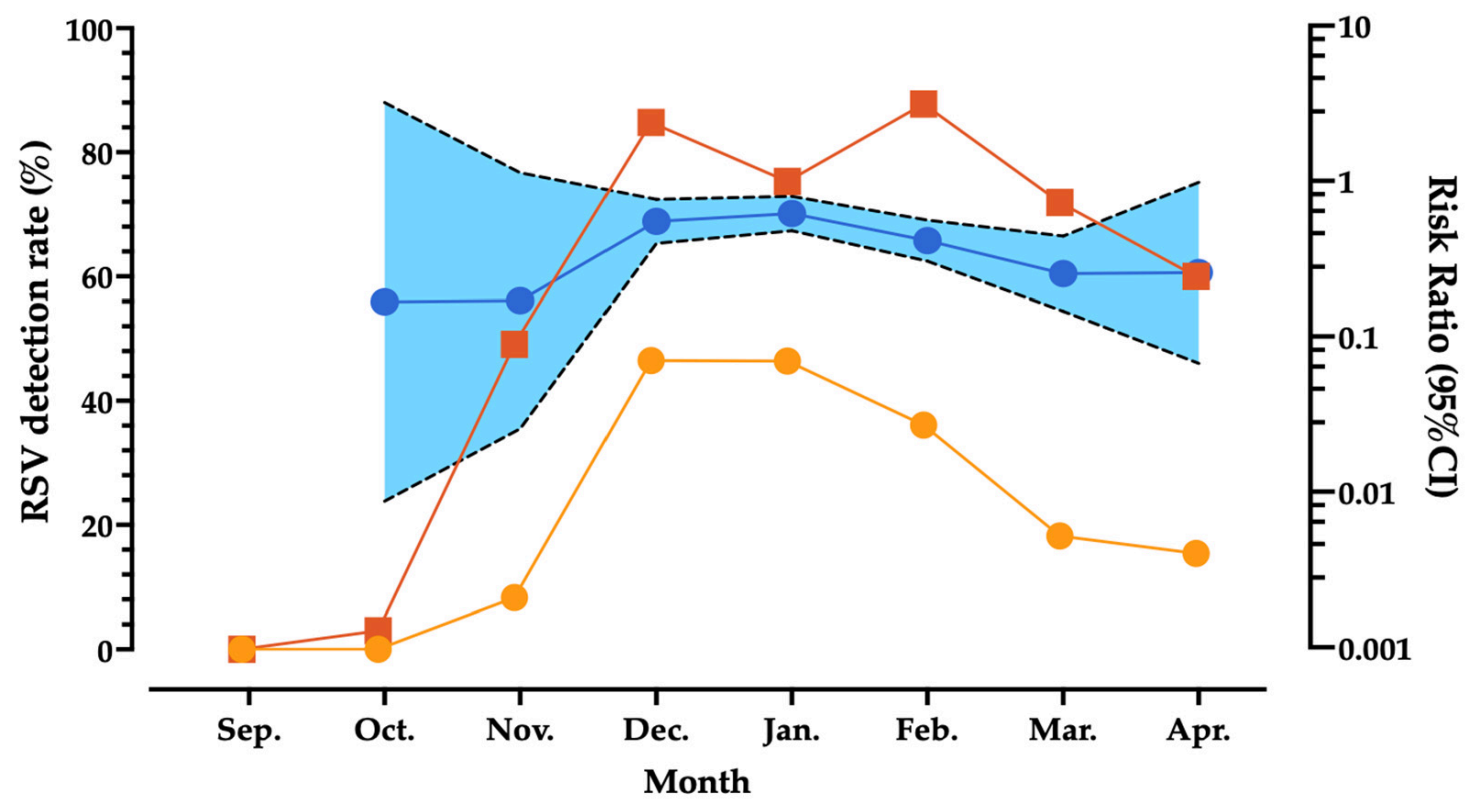
Monthly respiratory syncytial virus (RSV) detection rates among infants hospitalized for lower respiratory tract infection (LRTI), according to previous immunization with nirsevimab. Orange circles and red squares represent the descriptive monthly proportions of RSV-positive infants among nirsevimab-immunized and non-immunized hospitalized infants, respectively (left y-axis). Blue circles represent the corresponding monthly risk ratios (RRs) for RSV positivity comparing immunized with non-immunized infants, with the shaded area indicating the corresponding 95% confidence intervals (95% CIs; right logarithmic y-axis). Monthly RR estimates, particularly those from the peripheral months of the surveillance period, should be interpreted with caution because of the limited number of observations and the resulting imprecision of the estimates.

Across the epidemic season, a reduced risk of RSV infection associated with nirsevimab immunization was consistently observed during the core epidemic period, reaching statistical significance from December through March, with RRs ranging from 0.253 (95% CI 0.145–0.441) in March to 0.617 (95% CI 0.477–0.797) in January. Estimates for the peripheral months of the surveillance period were less precise and did not reach statistical significance (Figure 4).

Geographically, most cases originated from Southern Italy (45.45%), followed by Central Italy (23.08%), Northern Italy (19.81%), and the major islands of Sicily and Sardinia (11.67%) (Table 2). Infants from Northern Italy were not significantly older than those from the Major Islands (39.86 days ± 53.37 vs. 35.86 days ± 48.49, Kruskal–Walli’s test *p* = 0.775), but were significantly older than those of Central Italy (27.07 days ± 17.44; *p* = 0.009), and Southern Italy (30.02 ± 34.93; *p* = 0.026) (Appendix A Figure A4). The seasonal distribution of incident cases by region of origin, and particularly their proportional geographic distribution, suggested a North-to-South temporal gradient, as Northern Italy accounted for a larger proportion of cases during the early months of the study period, whereas the relative contribution of Southern Italy and the Major Islands progressively increased during the later months of the season (see Appendix A Figure A5).

Regarding the clinical severity of affected individuals, 63.91% of hospitalized infants required some form of respiratory support. Specifically, 45.59% required high-flow oxygen therapy, 27.68% required CPAP, and 0.95% required invasive respiratory support. NICU admission occurred in 19.67% of cases. The mean duration of hospitalization was 8.44 days ± 10.52 (median 7.00, IQR 4.00–9.00), with a right-skewed distribution (K2 test 1033, *p* < 0.001; Appendix A Figure A2B). Although LRTI-associated hospitalizations from Southern Italy were somewhat longer than those from Northern Italy (9.17 days ± 10.87 vs. 8.92 days ± 13.7), Central Italy (7.43 days ± 8.75) and the Major Islands (7.31 days ± 4.21), regional differences were not statistically significant (Kruskal–Wallis test, *p* value = 0.220; all post hoc tests, *p* > 0.05) (Appendix A Figure A4b).

No deaths were documented among the hospitalizations included in the analysis, and no correlation was identified between age at admission and length of hospital stay (Spearman’s rho = 0.047, 95% CI −0.028 to 0.121; Appendix A Figure A6).

### 3.2. Univariate Analysis

Comparisons of retrieved LRTI-associated hospitalizations by previous prophylaxis with nirsevimab and RSV infection status are reported in Table 3.

#### 3.2.1. Nirsevimab

**Figure 5:**
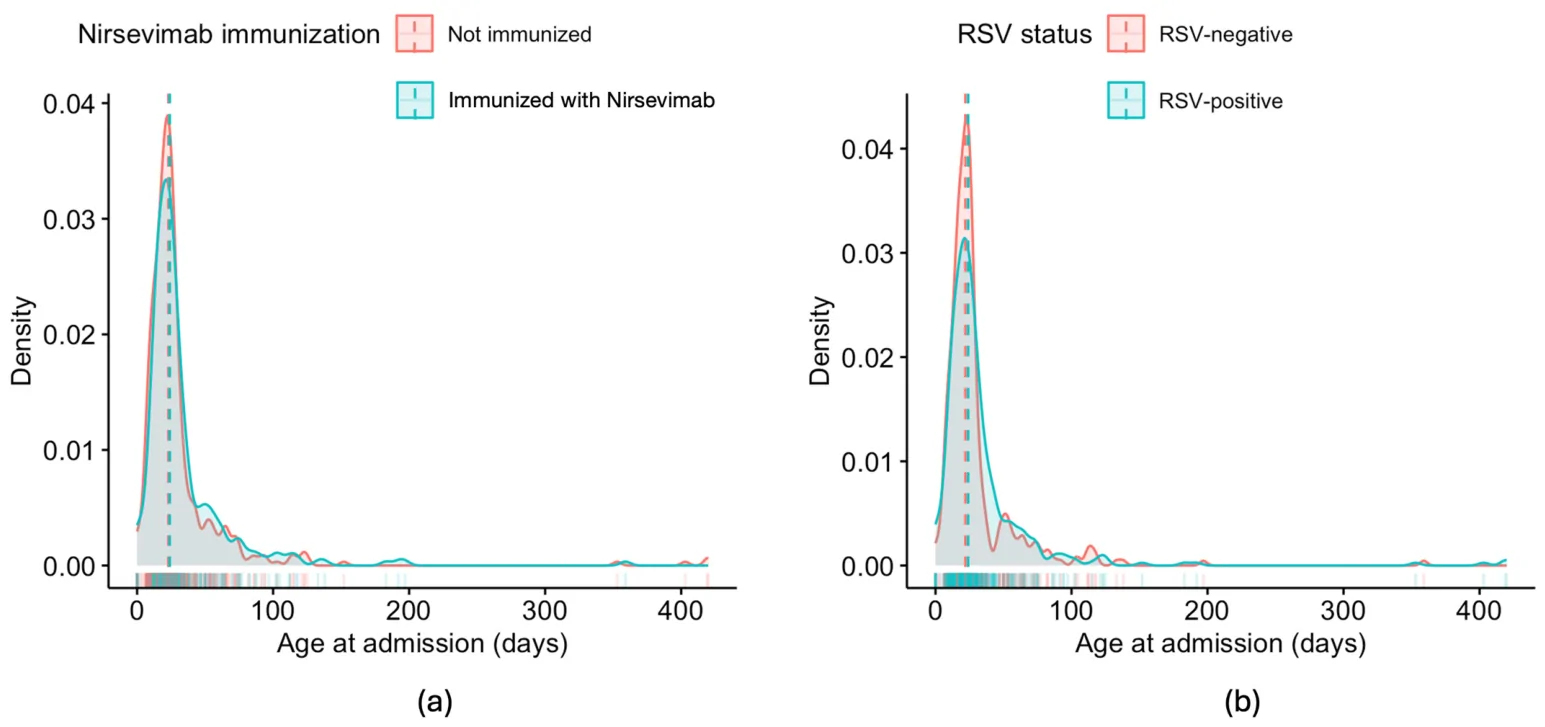
Distribution of age at admission according to (**a**) nirsevimab immunization status and (**b**) RSV status. Density curves illustrate the distribution of age at admission, with dashed vertical lines indicating group medians and rug plots representing individual observations.

As shown in Figure 5a, age at admission exhibited a markedly right-skewed distribution. Nevertheless, age distributions were largely overlapping between nirsevimab-immunized and non-immunized infants, with mean ages of 33.06 ± 33.53 and 31.20 ± 41.60 days, respectively (*p* = 0.517; SMD = 0.049). The median age at admission was 24.00 days (IQR 17.00–35.00) among nirsevimab-immunized infants and 23.00 days (IQR 16.00–30.00) among non-immunized infants.

Conversely, immunized infants were more frequently hospitalized between December and March than non-immunized infants (90.20% vs. 73.32%, *p* < 0.001; SMD = 0.448). The geographic distribution also differed between the two groups (*p* = 0.013; SMD = 0.244), with a higher proportion of immunized infants from Northern Italy (25.49% vs. 15.78%) and lower proportions from Central Italy (21.24% vs. 24.36%) and Southern Italy (41.83% vs. 48.03%). Comorbidities were more frequently reported among immunized than non-immunized infants (15.03% vs. 8.35%, *p* = 0.006; SMD = 0.209), particularly prematurity (10.13% vs. 5.57%, *p* = 0.029; SMD = 0.170). Conversely, documented RSV infection was substantially less frequent among immunized infants (33.99% vs. 68.21%, *p* < 0.001; SMD = 0.729).

Length of hospital stay was similar between immunized and non-immunized infants (7.76 ± 11.26 vs. 8.93 ± 9.96 days, respectively; *p* = 0.142; SMD = 0.093), as were NICU admission rates (20.59% vs. 19.03%, *p* = 0.666; SMD = 0.039). In contrast, infants who had received nirsevimab less frequently required respiratory support of any kind (55.23% vs. 70.07%, *p* < 0.001; SMD = 0.310) or CPAP support (18.95% vs. 33.87%, *p* < 0.001; SMD = 0.343), while a smaller difference was observed for high-flow oxygen therapy (40.85% vs. 48.96%, *p* = 0.036; SMD = 0.164). Invasive mechanical ventilation was also less frequent among immunized infants (0.65% vs. 1.16%), although this difference was not statistically significant (*p* = 0.754; SMD = 0.053).

#### 3.2.2. RSV Detection

RSV-positive and RSV-negative infants showed largely overlapping, markedly right-skewed age distributions (Figure 5b). The mean age at admission was 34.02 ± 44.34 days among RSV-positive infants and 29.57 ± 29.96 days among RSV-negative infants (*p* = 0.107; SMD = 0.117), while the corresponding median ages were 24.00 days (IQR 16.25–35.00) and 22.00 days (IQR 17.00–29.00), respectively. No significant differences in geographic distribution were identified according to RSV status (*p* = 0.617; SMD = 0.099). However, RSV-positive infants were substantially more likely to have been hospitalized between December and March than RSV-negative infants (90.20% vs. 69.32%, *p* < 0.001; SMD = 0.522). Previous nirsevimab prophylaxis was significantly less frequently reported among RSV-positive than among RSV-negative infants (26.13% vs. 59.59%, *p* < 0.001; SMD = 0.718).

In a separate exploratory analysis restricted to nirsevimab-immunized infants, we assessed whether the proportion of RSV-positive LRTI varied across age groups. Using infants aged 7–29 days as the reference group, a higher RR for RSV positivity was observed among those aged 30–59 days (RR 1.431, 95% CI 1.002–2.043). The corresponding RRs were 1.669 (95% CI 0.912–3.057) for infants aged <7 days, 1.406 (95% CI 0.796–2.483) for those aged 60–89 days, and 1.670 (95% CI 0.612–4.552) for those aged ≥90 days. These estimates describe age-related variation in RSV positivity among nirsevimab-immunized hospitalized infants and should not be interpreted as age-specific estimates of nirsevimab effectiveness.

Comorbidities were less frequently reported among RSV-positive than RSV-negative infants overall (7.29% vs. 15.63%, *p* = 0.001; SMD = 0.264), particularly prematurity (5.53% vs. 9.73%, *p* = 0.043; SMD = 0.159) and heart disease (0.75% vs. 4.72%, *p* = 0.002; SMD = 0.245).

RSV-positive infants showed longer hospital stays (median 8.00 days, IQR 6.00–10.00 vs. 5.00 days, IQR 3.00–8.00; *p* = 0.047; SMD = 0.143), more frequent requirement for respiratory support (77.89% vs. 47.49%, *p* < 0.001; SMD = 0.662), greater use of high-flow oxygen therapy (53.02% vs. 36.87%, *p* < 0.001; SMD = 0.329), and more frequent CPAP support (37.69% vs. 15.93%, *p* < 0.001; SMD = 0.507). Numerically higher proportions were also observed among RSV-positive infants for NICU admission (22.61% vs. 16.22%, *p* = 0.037; SMD = 0.162), while all seven cases requiring invasive mechanical ventilation occurred among RSV-positive infants (*p* = 0.038; SMD = 0.189). Given the exploratory nature of these univariate comparisons, these marginal findings should be interpreted cautiously.

### 3.3. Multivariable Analysis and Effectiveness Estimates

In the multivariable analysis (Table 4), when RSV-positive status was considered as the outcome variable (Model I), previous immunization with nirsevimab was independently associated with substantially reduced odds of RSV-associated LRTI (aOR 0.130, 95% CI 0.090–0.190). RSV positivity was strongly associated with hospitalization occurring between December and April (aOR 8.442, 95% CI 5.294–13.462), whereas lower odds were observed among infants with underlying risk factors (aOR 0.379, 95% CI 0.211–0.679). Compared with Northern Italy, lower odds of RSV positivity were observed among infants from Central Italy (aOR 0.581, 95% CI 0.345–0.976) and Southern Italy (aOR 0.476, 95% CI 0.301–0.752), while the difference for the Major Islands did not reach statistical significance (aOR 0.678, 95% CI 0.361–1.274). Age at admission was not significantly associated with RSV-positive status.

When the requirement for respiratory support was considered as the outcome (Model II), previous nirsevimab immunization was independently associated with reduced odds of requiring respiratory support (aOR 0.350, 95% CI 0.248–0.493). Respiratory support was more frequently required among infants hospitalized between December and April (aOR 2.670, 95% CI 1.770–4.029) and among those with underlying risk factors (aOR 3.464, 95% CI 1.757–6.828). Compared with Northern Italy, infants from Central Italy (aOR 0.319, 95% CI 0.188–0.541), Southern Italy (aOR 0.377, 95% CI 0.235–0.606), and the Major Islands (aOR 0.434, 95% CI 0.230–0.818) had significantly lower odds of requiring respiratory support. Age at admission was not significantly associated with this outcome.

Finally, when NICU admission was considered as the outcome (Model III), previous nirsevimab immunization was independently associated with reduced odds of NICU admission (aOR 0.441, 95% CI 0.233–0.837). No significant associations were identified for age at admission, hospitalization between December and April, or underlying risk factors. Conversely, compared with Northern Italy, substantially lower odds of NICU admission were observed among infants from Central Italy (aOR 0.081, 95% CI 0.042 to 0.158), Southern Italy (aOR 0.177, 95% CI 0.112 to 0.279), and the Major Islands (aOR 0.135, 95% CI 0.065 to 0.280).

### 3.4. Estimates of Immunization Effectiveness

**Figure 6:**
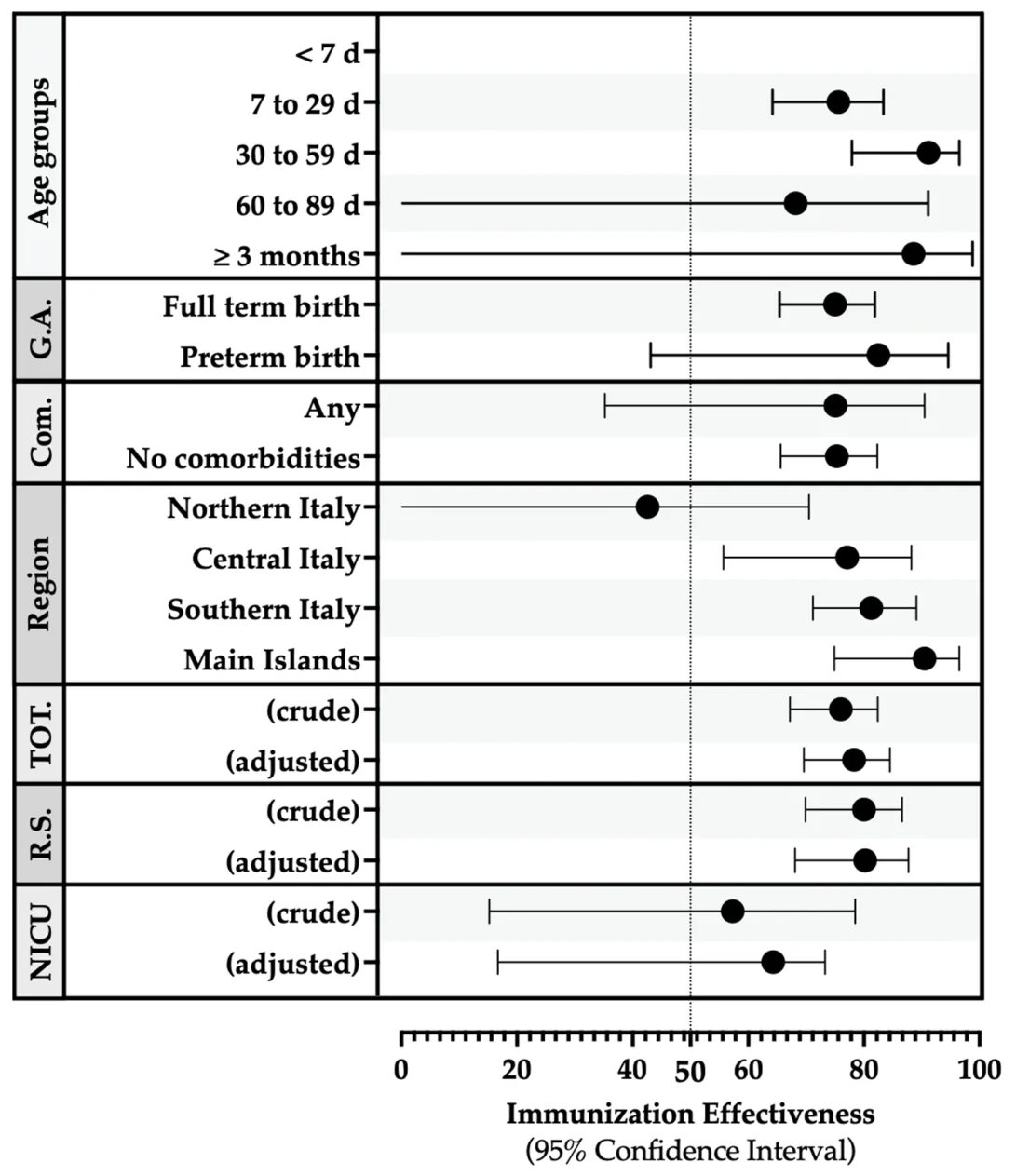
Estimates of immunization effectiveness by age groups, gestational age (GA), comorbidities (Com.), Region of origin, including the whole of retrieved subjects (TOT), by need for any respiratory support (RS), and by the need for neonatal intensive care unit (NICU) admission. Adjusted estimates were obtained by means of targeted maximum likelihood estimation, including in the model age at admission, prematurity, month of hospitalization, and geographical region. Error bars represent 95% confidence intervals.

Crude estimates. As shown in Figure 6 and Appendix A Table A3, IE against hospitalization due to RSV-associated LRTI was estimated at 76.0% (95% CI 67.2 to 82.4) for the whole sample in the univariate analysis.

In subgroup analyses, immunization effectiveness peaked in the Major Islands (90.5%, 95% 74.9 to 96.5), then decreased along a south-to-north gradient (Southern Italy, 81.3%, 95% CI 71.2 to 89.1; Central Italy, 77.1%, 95% CI 55.7 to 88.2; Northern Italy, 42.6%, 95% CI −11.8 to 70.5). Similar estimates were calculated for preterm (82.5%, 95% CI 43.1 to 94.6) and full-term infants (75.0%, 95% CI 65.4 to 81.9), and for infants with any reported comorbidity (75.1%, 95% CI 35.2 to 90.5) or without comorbidities (75.3%, 95% CI 65.6 to 82.3). Focusing on age at admission, no estimate was calculated for the age group < 7 days, while IE was estimated at 75.6% (95% CI 64.2 to 83.4) for the age group 7 to 29 days, peaking at 91.2% (95% CI 77.9 to 96.5) in the age group 30 to 59 days. Estimates of 68.2% (95% CI −13.5 to 91.1) and 88.6% (95% CI 10.8 to 98.8) were then calculated for older age groups of 60 to 89 days and ≥3 months, respectively. In secondary analyses based on 2 × 2 contingency tables among hospitalized infants, previous nirsevimab immunization was associated with relative reductions in the odds of respiratory support of 80.0% (95% CI 69.9–86.8) and NICU admission of 57.3% (95% CI 15.2–78.5), respectively. These estimates should be interpreted as associations within the hospitalized study population rather than as estimates of immunization effectiveness against these outcomes.

Multivariable estimates. Multivariable estimates were subsequently obtained using TMLE. For the primary outcome, TMLE yielded an estimated marginal probability of RSV positivity of 33.23% (95% CI 27.42–39.05) under nirsevimab immunization and 69.64% (95% CI 65.17–74.11) under no immunization. The resulting marginal odds ratio was 0.217 (95% CI 0.155–0.304), corresponding to an adjusted IE of 78.3% (95% CI 69.6–84.5%) within the test-negative design framework. The same TMLE framework was applied to the secondary clinical outcomes of respiratory support and NICU admission, using nirsevimab immunization as the exposure and the same set of adjustment covariates. Among hospitalized infants, the corresponding marginal odds ratios were 0.198 (95% CI 0.123–0.319) for respiratory support and 0.357 (95% CI 0.267–0.833) for NICU admission, corresponding to adjusted relative reductions in the odds of these outcomes of 80.2% (95% CI 68.1–87.7) and 64.3% (95% CI 16.7–73.3), respectively. These secondary estimates represent adjusted associations within the hospitalized study population and should not be interpreted as test-negative estimates of immunization effectiveness against these outcomes.

## 4. Discussion

### 4.1. Summary of Main Results

We report findings from a multicenter retrospective observational study involving 88 Italian pediatric centers, whose approximate potential catchment areas corresponded to 68.12% of the total Italian population aged <2 years at the beginning of the 2024–2025 winter season. Overall, the present study included 737 newborns and young infants hospitalized for LRTI, of whom 41.52% had previously received nirsevimab. A total of 398 infants (54.00%) had documented RSV infection, which was identified in 33.99% of immunized infants compared with 68.21% of non-immunized infants. Within the test-negative design framework, previous nirsevimab immunization was strongly associated with reduced odds of RSV positivity (aOR 0.130, 95% CI 0.090 to 0.190), while TMLE yielded an adjusted IE estimate of 78.3% (95% CI 69.6 to 84.5%) against RSV-associated hospitalization.

Secondary analyses of clinical severity among hospitalized infants showed comparable NICU admission rates according to immunization status (20.59% among nirsevimab recipients vs. 19.03% among non-immunized infants, *p* = 0.666), whereas immunized infants less frequently required respiratory support (55.23% vs. 70.07%, *p* < 0.001), particularly CPAP (18.95% vs. 33.87%, *p* < 0.001), with a smaller difference observed for high-flow oxygen therapy (40.85% vs. 48.96%, *p* = 0.036). In the corresponding TMLE analyses, previous nirsevimab immunization was associated with adjusted relative reductions in the odds of respiratory support and NICU admission of 80.2% (95% CI 68.1–87.7%) and 64.3% (95% CI 16.7–73.3%), respectively. Unlike the primary test-negative estimate, these secondary findings represent adjusted associations within the hospitalized study population and should be interpreted as exploratory rather than as direct estimates of immunization effectiveness against clinical severity.

### 4.2. Generalizability of Main Results

The main finding of this study is the markedly lower proportion of laboratory-confirmed RSV infection among nirsevimab-immunized infants hospitalized for LRTI. Within the test-negative design, this finding was consistent with a substantial protective effect of nirsevimab against RSV-associated hospitalization. This association remained evident after multivariable adjustment and in the TMLE analysis, in which prior immunization was associated with a lower likelihood of RSV-positive hospitalization. These observations are consistent with the efficacy reported in pivotal clinical trials [38,58,59,60,61] and with the growing body of real-world evidence from European and North American settings [48,49,53,54,55,56,62,63,64,65,66,67], supporting the effectiveness of nirsevimab in preventing severe RSV disease during infancy [66,68,69,70]. Subgroup analyses further support the consistency of the protective effect of nirsevimab. Effectiveness estimates against RSV-associated hospitalization remained high in both full-term and preterm infants [71] and were particularly notable in infants aged 30–59 days. Although estimates in some smaller subgroups were characterized by wide confidence intervals, likely due to limited sample size, the overall direction of effect remained consistent. This is clinically relevant because it suggests that nirsevimab may be beneficial not only in traditionally high-risk categories but also in the broader population of otherwise healthy young infants, who represented most RSV-positive hospitalizations in our study population.

A second relevant finding concerns disease severity. Although the duration of hospitalization (7.76 days ± 11.26 vs. 8.93 days ± 9.96, *p* = 0.142) and the frequency of NICU admission (20.59% vs. 19.03%, *p* = 0.666) did not differ significantly between immunized and non-immunized infants in unadjusted comparisons. In outcome-specific multivariable analyses, previous nirsevimab administration was strongly associated with a reduced likelihood of RSV-positive hospitalization after adjustment for age at admission, month of hospitalization, comorbidities, and geographic region (aOR 0.130, 95% CI 0.090 to 0.190), supporting the robustness of the association observed in the test-negative analysis (Model I). When clinical severity was considered separately, nirsevimab prophylaxis was also independently associated with a substantially lower likelihood of requiring respiratory support in Model II (aOR 0.350, 95% CI 0.248 to 0.493). An inverse association was likewise observed for NICU admission in Model III (aOR 0.441, 95% CI 0.233 to 0.837). Taken together, these outcome-specific models suggest that the association with nirsevimab was not restricted to a lower probability of RSV-positive hospitalization, but extended to clinically relevant indicators of disease severity among hospitalized infants. Nevertheless, given the observational nature of the study and the potential influence of residual confounding, differences in admission practices, and other unmeasured determinants of clinical severity, these findings should be interpreted as adjusted associations rather than evidence of a direct causal effect of nirsevimab on the subsequent clinical course.

**Figure A1:**
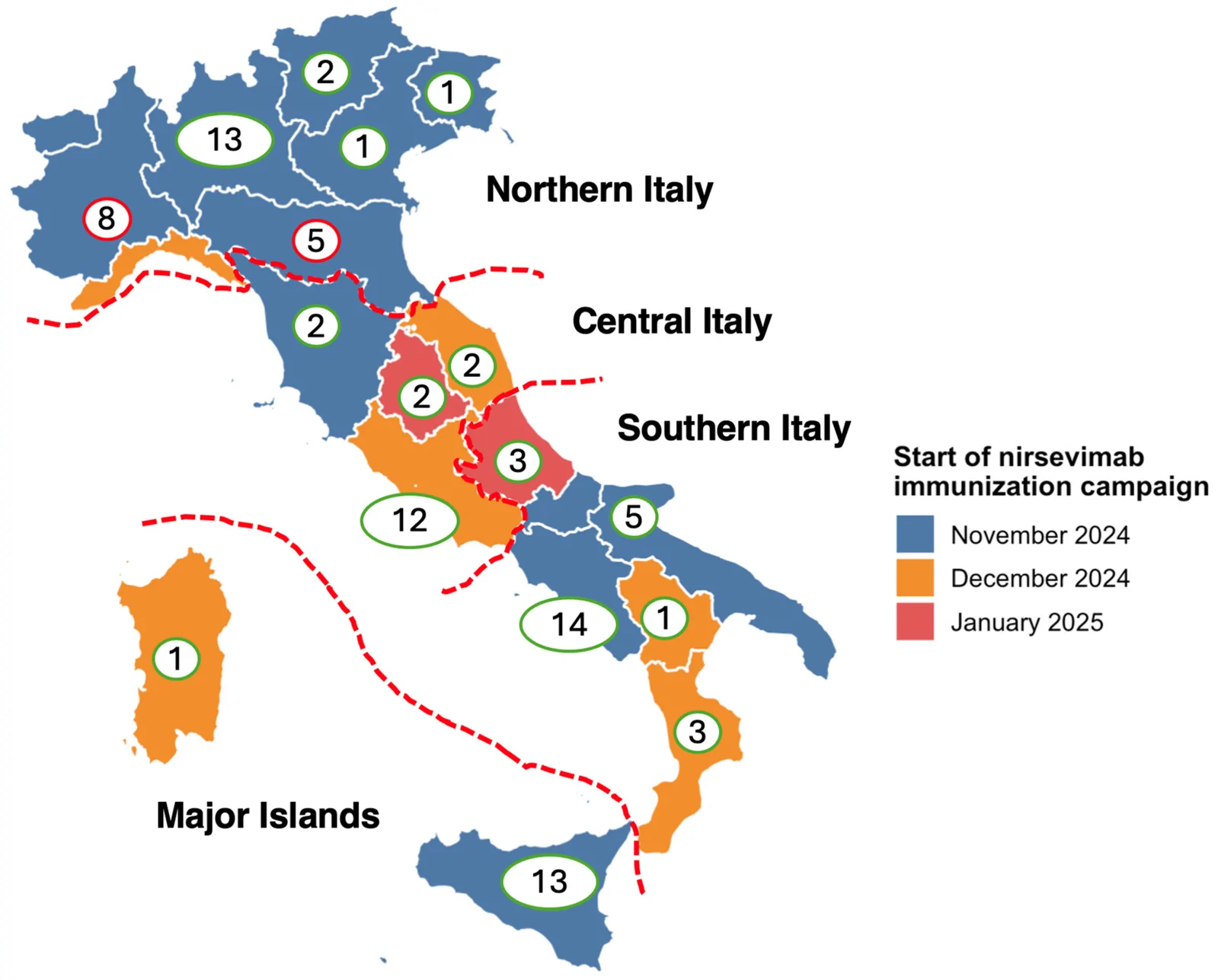
Regional timing of nirsevimab implementation during the 2024–2025 RSV season in Italy and number of participating centers from each Region. Regions are classified according to the month of initiation of the nirsevimab immunization campaign (November 2024, December 2024, or January 2025). The Autonomous Provinces of Trento and Bolzano were considered jointly as a single geographical unit. Dashed lines represent the borders between main Italian areas (i.e., Northern Italy, Central Italy, Southern Italy, and Major Islands).

**Figure A2:**
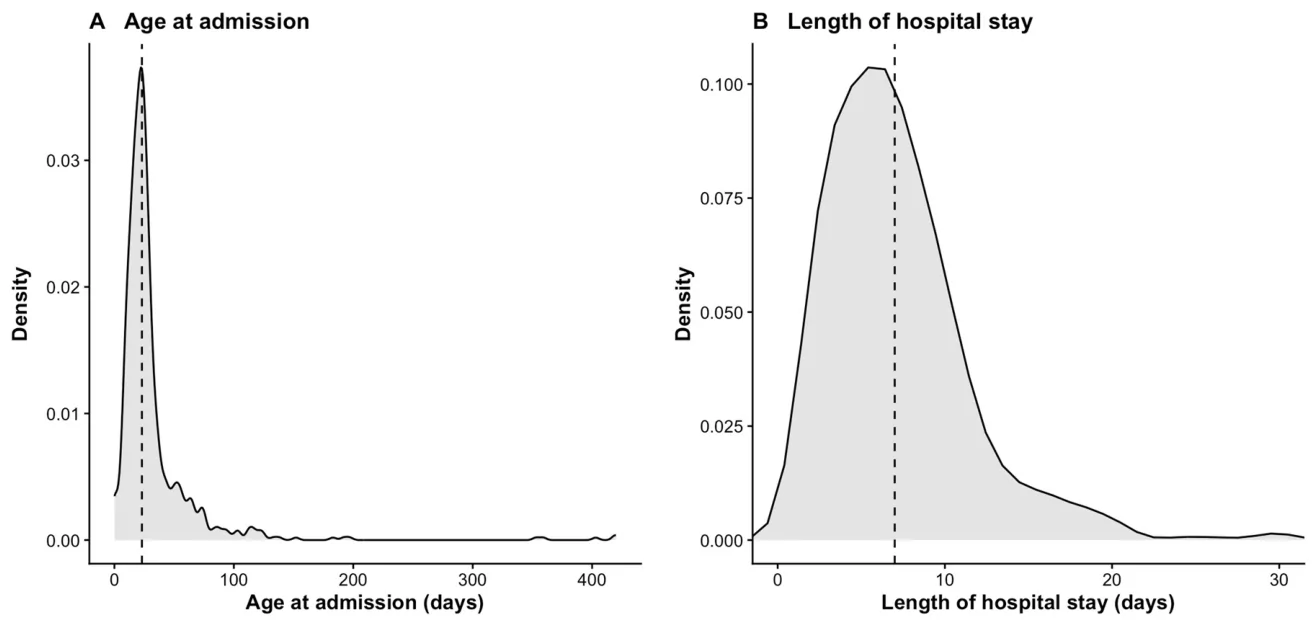
Distribution of sampled cases of hospitalizations due to lower respiratory tract infections by their age at admission (**A**) and hospital stay (**B**). Dashed vertical lines indicate group medians and rug plots representing individual observations.

**Figure A4:**
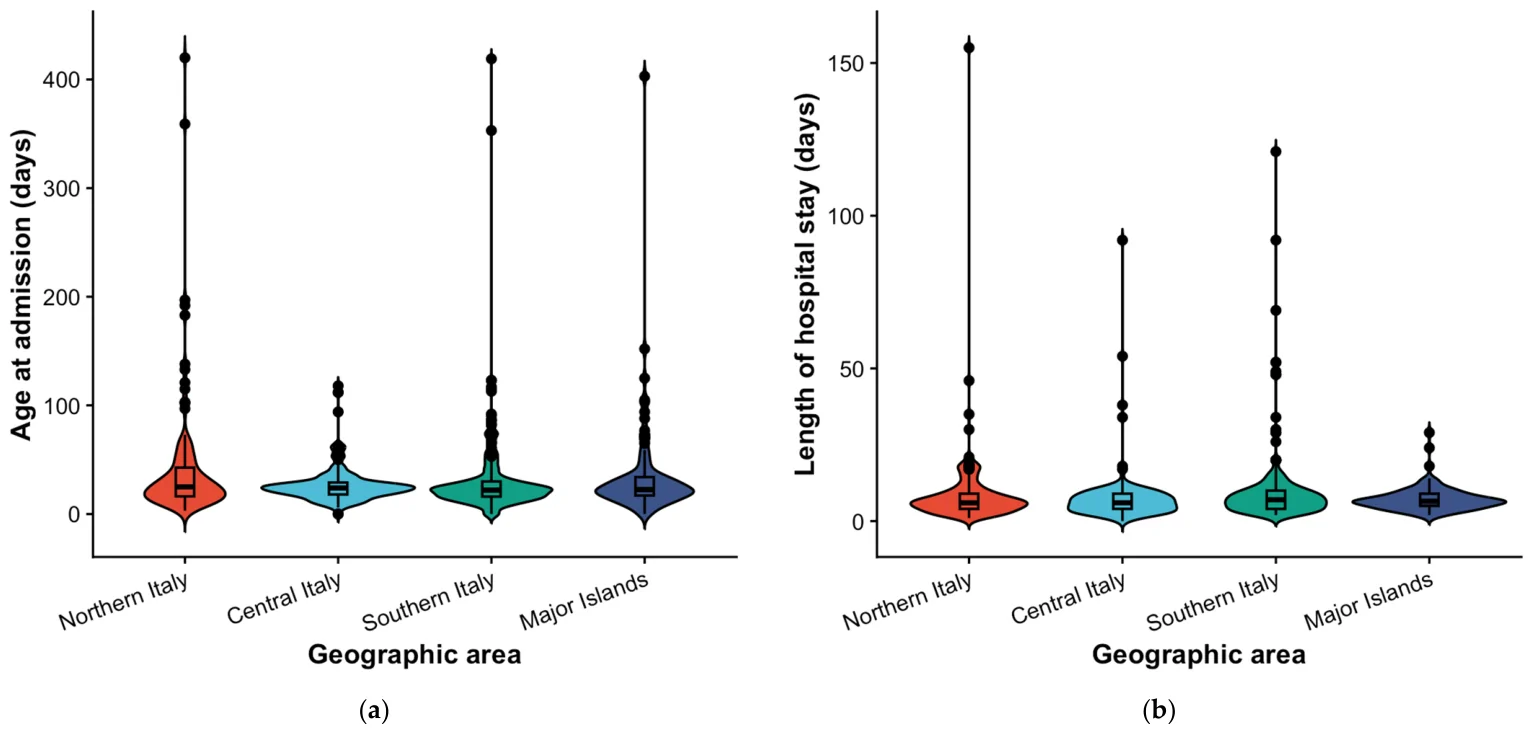
Distribution of sampled cases of hospitalizations due to lower respiratory tract infections by geographic area according to their age at admission (**a**) and their hospital stay (**b**).

**Figure A5:**
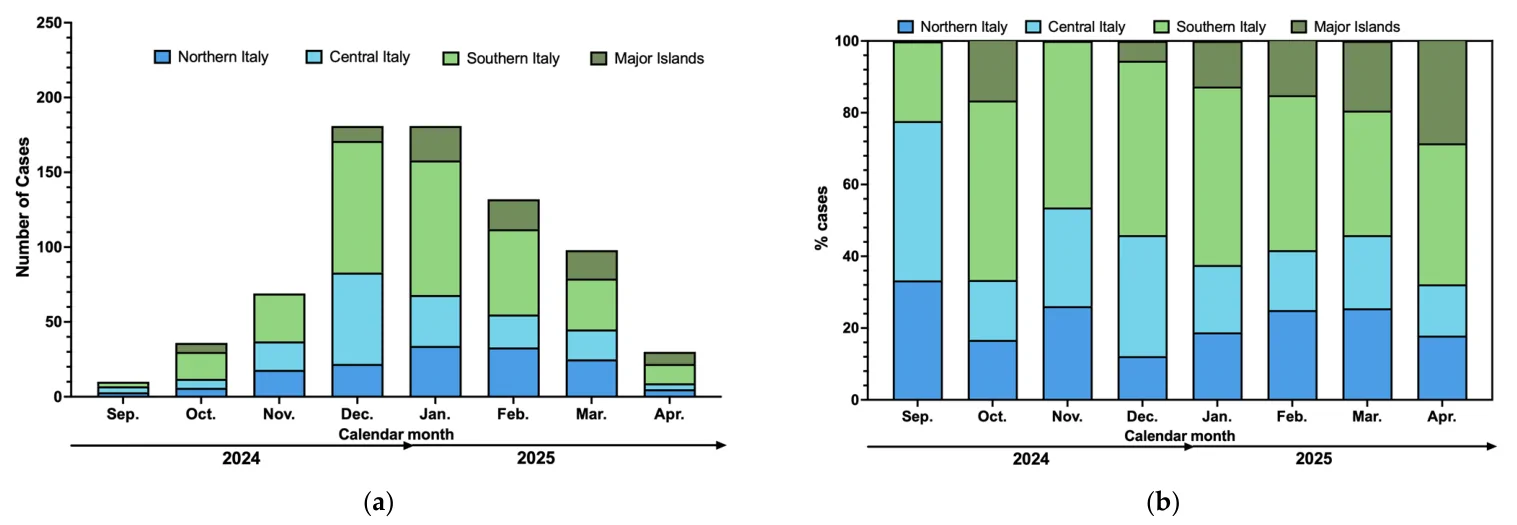
Distribution of sampled cases of hospitalizations due to lower respiratory tract infections by region of origin and calendar month of hospital admission, reported in crude numbers (**a**) and as percent estimate of monthly hospitalizations (**b**).

**Figure A6:**
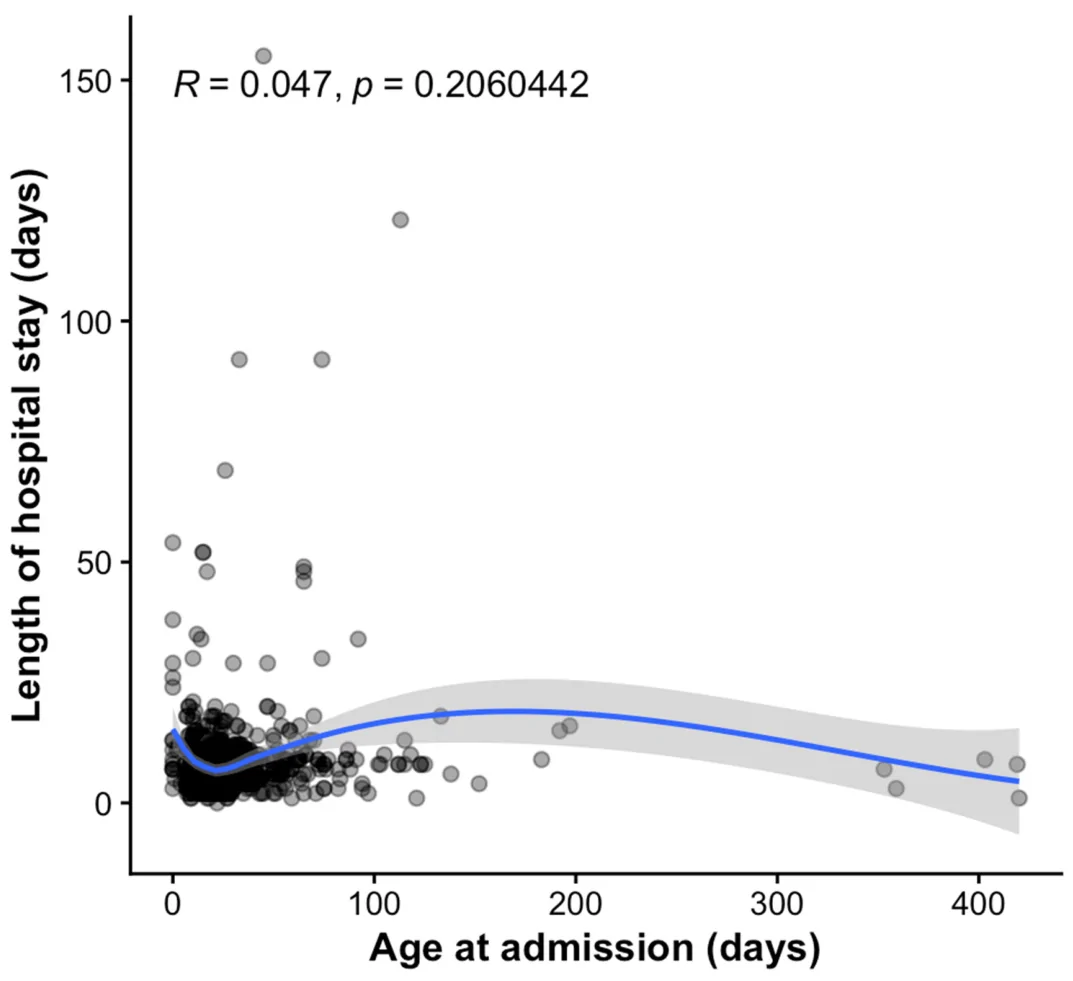
Relationship between age at admission and length of hospital stay. Individual observations are shown as points, while the solid line represents the locally estimated scatterplot smoothing (LOESS) curve with its 95% confidence interval. The association between age and length of hospital stay was assessed using Spearman’s rank correlation coefficient (Spearman’s rho = 0.047, 95% CI −0.028 to 0.121).

Again, the present results are highly consistent with previous reports [72,73,74,75,76], including the multicenter case–control studies by Zambrano et al. [73] and Moline et al. [77]. Still, it should be stressed that while the overall estimates against RSV-associated hospitalization are in line with the currently available real-world evidence [49,78], the adjusted association between previous nirsevimab immunization and NICU admission observed in our hospitalized study population was weaker than the effectiveness estimates against NICU admission reported by Zambrano et al. (i.e., 80%, 95% CI 70 to 86) and Moline et al. (89.4%, 95% CI 67.2 to 96.6) [73,77]. Several explanations may account for this difference. First, hospitalization policies and admission practices within a universal healthcare system such as the Italian National Health Service may not be directly comparable with those adopted in other countries, particularly in insurance-based healthcare systems such as that of the United States [79,80,81,82,83]. Second, as previously stressed [56,84], the implementation of universal nirsevimab prophylaxis during the first RSV season in 2024–2025 in Italy was highly heterogeneous across the various regional authorities (see Appendix A Figure A1), a pattern also documented in previous reports [53,54,62,84] and reflected in our data. In fact, when crude IE was estimated by geographic area, Northern Italy was characterized by lowest estimate (IE 42.6%, 95% −11.8 to 70.5) compared with other geographic areas (Central Italy: 77.1%, 95% CI 55.7 to 88.2; Southern Italy 81.3%, 95% CI 71.2 to 89.1; Major Islands 90.5%, 95% CI 74.9 to 96.5; see also Appendix A Table A3). Third, the temporal distribution of cases also suggested a geographical North-to-South gradient across the RSV season, with an earlier relative predominance of hospitalizations in Northern Italy followed by an increasing contribution from Southern Italy and the Major Islands as the season progressed (Appendix A Figure A4). This pattern may, in turn, have influenced the estimates because of heterogeneous availability of high-intensive healthcare options. Fourth, as shown in Appendix A Table A4, NICU utilization across the various Italian regions was also markedly heterogeneous. More precisely, 49.3% of LRTI cases from Northern Italy were admitted to NICU, compared with 7.6% from Central Italy, 14.6% from Southern Italy, and 12.8% from Main Islands, with corresponding RRs consistently lower than that for Northern Italy (i.e., RR 0.155, 95% CI 0.090 to 0.268 for Central Italy; RR 0.297, 95% CI 0.219 to 0.403 for Southern Italy RR 0.259, 95% CI 0.146 to 0.461 for the Major Islands). As also suggested by the data on respiratory support, we cannot rule out the possibility that participating centers from Northern Italy, which included a high proportion of tertiary-level NICUs, might have oversampled more severe cases compared with other regions, leading to a potential overestimation of the NICU admission rate in Northern Italy and thereby influencing the magnitude of the observed association between nirsevimab immunization and reduced NICU admission.

In other words, our results are consistent with available national [51,52,53,54,55,62,63] and international reports suggesting that, beyond reducing the occurrence of RSV-associated hospitalization, nirsevimab may also be associated with lower clinical severity among breakthrough infections, as recently reported in other studies [44,67,72,73,77]. From a clinical perspective, a lower requirement for respiratory support is particularly meaningful in early infancy, when respiratory deterioration may occur rapidly and often requires close monitoring, higher-intensity care, and increased healthcare resource utilization.

**Figure A7:**
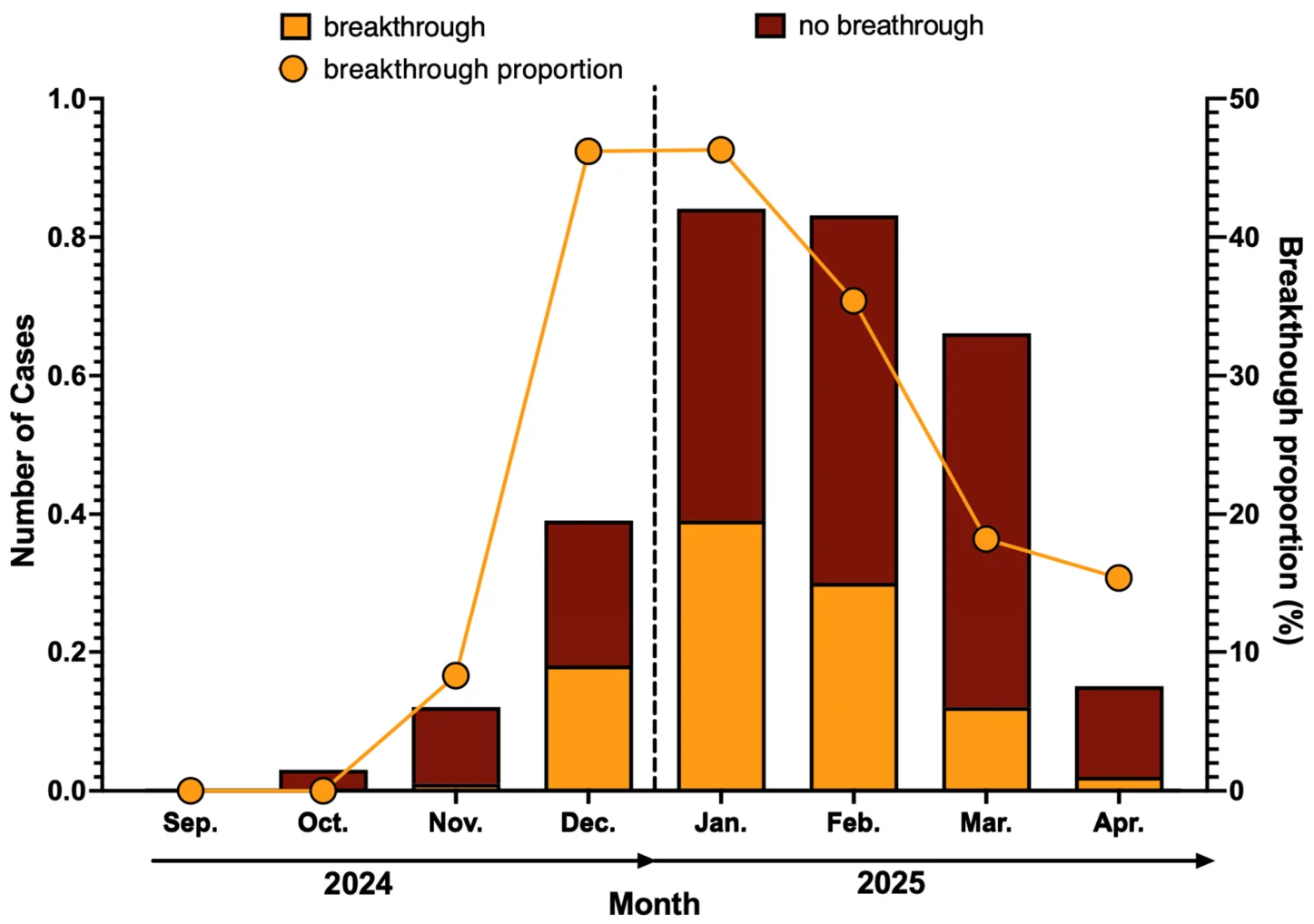
Distribution of breakthrough cases of respiratory syncytial virus infections in children immunized by means of nirsevimab monoclonal antibody.

Third, our findings confirm the major contribution of RSV to severe respiratory morbidity in the first months of life [4,85,86,87], not only among non-immunized infants, in whom RSV accounted for 68.21% of total cases, but also among immunized infants, as previously reported in several Italian studies [51,56,63]. In fact, more than half of the hospitalized infants tested positive for RSV, while RSV positivity was documented in 33.99% of previously immunized infants. Among immunized infants, RSV positivity showed an age-dependent distribution and a seasonal pattern, with higher proportions during the first weeks of life and in December 2024 and January 2025 (Appendix A Figure A7). Again, these results may be explained by differences in the implementation strategies for universal immunization in Italy and in catch-up campaigns, where implemented [49,84,88]. As highlighted by Badolato et al. [88], the initial implementation of the universal nirsevimab immunization campaign was not only heterogeneous across Italian regions (Appendix A Figure A1), but substantial variability was also observed in the adoption and organization of catch-up strategies, where implemented (see Appendix A Table A5), leading to potential heterogeneity in RSV infection rates across the populations of the regions in which the participating centers were located. In other words, our results from a broad multicenter and multiregional Italian sample indicate that the association between previous nirsevimab immunization and lower RSV positivity was observed despite heterogeneous implementation conditions across regions, which may nevertheless have influenced the magnitude of the corresponding estimates.

Another relevant aspect of the present study is the seasonal pattern of admissions and RSV detection. Despite geographic variability in seasonal patterns across the Italian Peninsula due to heterogeneous environmental factors, the great majority of hospitalizations occurred between December and March, and RSV detection rates peaked in December, in line with the expected winter epidemic pattern in Italy [88,89]. Notably, throughout the season, the proportion of RSV-positive cases remained consistently lower among immunized infants. This descriptive consistency in the direction of the association across the months of highest viral circulation is reassuring; however, given the unequal monthly numbers of immunized and non-immunized infants and the geographic and temporal heterogeneity of nirsevimab implementation, it should not be interpreted as evidence of temporal stability of immunization effectiveness [90,91].

### 4.3. Limitations and Strengths of the Present Study

#### 4.3.1. Limitations

Despite its potential significance for clinical practice and public health policy, some limitations should be acknowledged. First, the retrospective observational design precludes full control of confounding and does not allow causal inference to the same extent as randomized studies. Although we adjusted for key baseline variables, residual confounding cannot be excluded [92,93,94].

Second, the study included only hospitalized infants evaluated in neonatal settings, with a possible overrepresentation of secondary- and tertiary-level centers, particularly from certain regions. Therefore, the findings may not be fully generalizable to milder community-managed infections or to admissions occurring in pediatric wards outside the participating network, and selection bias arising from conditioning study inclusion on hospitalization for LRTI cannot be excluded. The characteristics of participating centers may also have influenced clinical outcomes, particularly NICU admission rates. In addition, RSV testing was performed according to local diagnostic protocols using either molecular or antigen-based assays, and some degree of between-center heterogeneity in diagnostic sensitivity cannot be excluded. However, the predefined case definition and standardized data collection procedures ensured reasonable consistency in the clinical information collected across centers.

Third, regional variability in campaign implementation, timing of administration, access to prophylaxis, and catch-up strategies may have affected immunization coverage during the epidemic season [56,84,88]. Differences in healthcare-seeking behavior, hospital admission thresholds, regional availability and timing of nirsevimab prophylaxis, and local RSV testing practices may therefore have influenced the composition of the hospitalized study population.

Fourth, some methodological concerns must be addressed. Some subgroup analyses were based on relatively small numbers and should therefore be interpreted with caution. Multiple univariate comparisons were also performed for exploratory characterization of the study population, without formal adjustment for multiplicity. Accordingly, isolated marginal *p* values, particularly those close to the conventional 0.05 threshold, should be interpreted cautiously and not regarded as independent confirmatory evidence. The TMLE implementation also used a Super Learner library restricted to SL.glm and SL.mean and therefore provided limited algorithmic diversity, potentially reducing some of the advantages of ensemble-based nuisance parameter estimation. Formal diagnostics of the positivity assumption were not performed and, given the substantial temporal and geographic heterogeneity in nirsevimab implementation across Italy, limited exposure overlap within some combinations of month and geographic region cannot be excluded. TMLE estimates should therefore be interpreted in light of these limitations.

Fifth, the original study design did not require participating centers to systematically report pathogens other than RSV. Among the 339 RSV-negative LRTI hospitalizations, information on alternative respiratory pathogens was available for only 87 cases (25.66%), limiting our ability to determine whether the seasonal pattern observed for RSV was accompanied by changes in other respiratory infections [56,57,95,96]. The available information on other respiratory pathogens is nevertheless reported in Appendix A Table A6.

Sixth, reliable information on the number of nirsevimab doses administered during the study period was available only for selected Italian regions [41,70,90]. Therefore, the resulting estimates of immunization effectiveness should be extrapolated to the general pediatric population with considerable caution. Furthermore, complete information on LRTI hospitalizations, and particularly RSV-associated LRTI hospitalizations, during previous epidemic seasons could not be retrieved from all participating centers. Consequently, the present study could not directly estimate the impact of the immunization campaign on hospitalization rates compared with previous seasons. Historical data available from other Italian studies nevertheless suggest a substantial reduction in RSV-associated hospitalizations following nirsevimab implementation [52,55,97,98,99,100].

Seventh, several potentially relevant individual and environmental factors could not be assessed. These included not only relevant demographic data (i.e., parental ethnicity, socioeconomic status, and municipality of residence) but also environmental and microclimatic exposures, outdoor and indoor air pollutants, including PM10, PM2.5, and tobacco smoke [101,102,103,104,105] as well as parental ethnicity, socioeconomic status, and municipality of residence. The absence of this information prevented the inclusion of these factors as potential covariates in the analyses.

Eight, owing to constraints related to current Italian privacy regulations and the requirements governing the use of the data included in this study, the exact date of nirsevimab administration was unavailable, limiting the interpretation of breakthrough infections, for which an adequate post-administration protection interval could not be established.

Finally, the study focused on short-term hospital outcomes and did not assess longer-term respiratory sequelae, and it provides an estimate of the direct and indirect costs borne by families during hospitalization and its aftermath. Given the potential medium- and long-term impact of severe RSV infection on pediatric respiratory health [106,107,108], as well as its possible neurological and cardiovascular complications [61,109,110,111], the present findings may provide useful evidence to inform future cost-effectiveness analyses, but cannot themselves be regarded as a cost-effectiveness assessment.

#### 4.3.2. Strengths

Despite these limitations, the present findings have important public health implications. In a setting where RSV still places a substantial burden on neonatal and pediatric services every winter, an intervention capable of reducing RSV-associated hospitalization may translate into meaningful benefits for infants, families, and healthcare systems. Moreover, the lower requirement for respiratory support observed among previously immunized infants in our hospitalized cohort is particularly relevant in periods of seasonal service pressure, when preventing escalation of care may help preserve intensive and sub-intensive care capacity.

An important strength of this study lies in its real-world multicenter design and its broad multicenter and multiregional representation, involving numerous neonatal units distributed across different Italian geographical areas. This allowed us to capture routine clinical practice during the first season of universal implementation and to evaluate outcomes in a population that reflects actual healthcare delivery rather than the highly selected populations typically enrolled in randomized controlled trials. In addition, the use of laboratory-confirmed RSV diagnosis strengthens the specificity of the outcome definition. The application of multivariable analysis and TMLE allowed adjustment for measured confounding and provided an analytical framework less dependent on the specification of a single conventional regression model. However, these methods cannot eliminate residual or unmeasured confounding, selection bias, or potential violations of the positivity assumption.

### 4.4. Implications for Medical Practice

Overall, this study strengthens the real-world evidence supporting the effectiveness of nirsevimab against RSV-associated hospitalizations among newborns and young infants in the first epidemic season [49,71,78]. Within the hospitalized study population, previous nirsevimab immunization was also associated with a lower requirement for respiratory support, although these secondary findings should be interpreted as adjusted associations rather than as direct estimates of effectiveness against clinical severity. The present findings provide evidence from a broad multicenter and multiregional setting in which implementation varied geographically and temporally across participating areas. Future studies should further assess the impact of nirsevimab over multiple seasons, explore regional differences in implementation and coverage, and evaluate broader outcomes, including healthcare utilization, cost-effectiveness, and long-term respiratory morbidity. Nevertheless, the present data support the role of nirsevimab as an important preventive tool against RSV disease in early infancy.

## 5. Conclusions

This multicenter study provides real-world evidence that previous nirsevimab immunization is associated with substantially lower odds of RSV-positive hospitalization among newborns and young infants within a test-negative design framework. Among infants hospitalized for LRTI, previous niservimab immunization was also associated with a lower requirement for respiratory support, particularly high-flow oxygen therapy and CPAP.

These findings support the real-world effectiveness of nirsevimab against RSV-associated hospitalization within the hospitalized test-negative study population, while the observed associations with clinical severity outcomes should be interpreted as associations within the selected hospitalized population. The present findings are also consistent with the reduction in healthcare burden documented by population-based studies. Widespread implementation of nirsevimab may contribute to reducing the healthcare burden associated with seasonal RSV epidemics.

## Figures and Tables

**Table 1:** Characteristics of the potential catchment population.

	Age Group (No.)	
	<2 Years of Age	<1 Year of Age
Italian General Population	731,314	358,173
Total Potential catchment population	509,244	249,960
% of Italian General Population	69.63%	69.79%

**Table 2:** Main characteristics of newborns and infants enrolled in the survey (note: SD = standard deviation; LRTI = lower respiratory tract infection; RSV = respiratory syncytial virus; CPAP = Continuous Positive Airway Pressure; NICU = neonatal intensive care unit).

Variable	Total (N/737, %)
Age at admission (days, average ± SD)	31.97 ± 38.44
Month of diagnosis	
September to November	145, 19.67%
December to April	592, 80.32%
Area of origin	
Northern Italy	146, 19.81%
Central Italy	170, 23.08%
Southern Italy	335, 45.45%
Main Islands	86, 11.67%
Prophylaxis with nirsevimab	306, 41.52%
RSV-associated LRTI	398, 54.00%
Comorbidities	
Any	82, 11.13%
Prematurity	55, 7.46%
Heart disease	19, 2.58%
Lung disease	4, 0.54%
Immune depression	2, 0.27%
Trisomy 21	2, 0.27%
Need for respiratory support	
Any	471, 63.91%
O_2_ high flow	336, 45.59%
CPAP	204, 27.68%
Invasive Respiratory support	7, 0.95%
NICU admission	145, 19.67%
Mortality	-
Hospital stay (days, average ± SD)	8.44 ± 10.52

**Table 3:** Main characteristics of newborns and infants enrolled in the stratified by Respiratory Syncytial Virus (RSV) infection status (note: SD = standard deviation; LRTI = lower respiratory tract infection; CPAP = Continuous Positive Airway Pressure; NICU = neonatal intensive care unit).

Variable	Prophylaxis with Nirsevimab		SMD	*p* Value ^1^	RSV-Positive Status		*SMD*	*p* Value ^1^
	Yes (N/306, %)	No (N/431, %)			Yes (N/398, %)	No (N/339, %)		
Age at admission (days)			0.049	0.517			0.117	0.107
average ± SD	33.06 ± 33.53	31.20 ± 41.60			34.02 ± 44.34	29.57 ± 29.96		
Median, interquartile range	24.00 [17.00; 35.00]	23.00 [16.00; 30.00]			24.00 [16.25; 35.00]	22.00 [17.00; 29.00]		
Month of diagnosis			0.448	< 0.001			0.522	<0.001
September to November	30, 9.80%	115, 26.68%			30, 9.80%	104, 30.68%		
December to April	276, 90.20%	316, 73.32%			276 90.20%	235, 69.32%		
Area of origin			0.244	0.013			0.099	0.617
Northern Italy	78, 25.49%	68, 15.78%			84, 21.11%	62, 18.29%		
Central Italy	65, 21.24%	105, 24.36%			92, 23.12%	78, 23.01%		
Southern Italy	128, 41.83%	207, 48.03%			173, 43.47%	162, 47.79%		
Main Islands	35, 11.44%	51, 11.83%			49, 12.31%	37, 10.91%		
Prophylaxis with nirsevimab	-	-		-	104, 26.13%	202, 59.59%	0.718	<0.001
RSV-associated LRTI	104, 33.99%	294, 68.21%	0.729	<0.001	-	-		-
Comorbidities								
Any	46, 15.03%	36, 8.35%	0.209	0.006	29, 7.29%	53, 15.63%	0.264	0.001
Prematurity	31, 10.13%	24, 5.57%	0.170	0.029	22, 5.53%	33, 9.73%	0.159	0.043
Heart disease	10, 3.27%	9, 2.09%	0.073	0.447	3, 0.75%	16, 4.72%	0.245	0.002
Lung disease	3, 098%	1, 0.23%	0.097	0.393	0, -	4, 1.18%	0.155	0.095
Immune depression	1, 0.33%	1, 0.23%	0.018	1.000	2, 0.50%	0, -	0.101	0.551
Down Syndrome	1, 0.33%	1, 0.23%	0.018	1.000	2, 0.50%	0, -	0.101	0.551
Need for respiratory support								
Any	169, 55.23%	302, 70.07%	0.310	<0.001	310, 77.89%	161, 47.49%	0.662	<0.001
O_2_ high flow	125, 40.85%	211, 48.96%	0.164	0.036	211, 53.02%	125, 36.87%	0.329	<0.001
CPAP	58, 18.95%	146, 33.87%	0.343	<0.001	150, 37.69%	54, 15.93%	0.507	<0.001
Invasive Respiratory support	2, 0.65%	5, 1.16%	0.053	0.754	7, 1.76%	0, -	0.189	0.038
NICU admission	63, 20.59%	82, 19.03%	0.039	0.666	90, 22.61%	55, 16.22%	0.162	0.037
Mortality	-	-	-	-	-	-	-	-
Hospital stay (days)								
average ± SD	7.76 ± 11.26	8.93 ± 9.96	0.093	0.142	9.16 ± 9.90	7.61 ± 11.22	0.143	0.047
Median, interquartile range	6.00 [4.00; 9.00]	7.00 [5.00; 10.00]			8.00 [6.00; 10.00]	5.00 [3.00; 8.00]		

^1^ Mann–Whitney test for continuous data; chi-squared test for categorical data.

**Table 4:** Association of RSV-positive status among children hospitalized because of lower respiratory tract infection (Model I), need for any respiratory support (Model II), and Neonatal Intensive Care Unit (NICU) admission (Model III) with main demographic and outcome variables. Adjusted Odds Ratios (aOR) with their corresponding 95% confidence intervals (95% CI) were calculated by means of binary logistic regression analysis.

Condition	RSV-Associated LRTI (Model I)	Respiratory Support (Model II)	NICU Admission (Model III)
	aOR (95% CI)	aOR (95% CI)	aOR (95% CI)
Age at admission	1.004 (0.998; 1.010)	1.008 (1.000; 1.015)	1.003 (0.998; 1.007)
Hospital stay	-	-	-
Admission: December to April	8.442 (5.294; 13.462)	2.670 (1.770; 4.029)	1.455 (0.849; 2.395)
Region of Origin			
Northern Italy	1.000 (REFERENCE)	1.000 (REFERENCE)	1.000 (REFERENCE)
Central Italy	0.581 (0.345; 0.976)	0.319 (0.188; 0.541)	0.081 (0.042; 0.158)
Southern Italy	0.476 (0.301; 0.752)	0.377 (0.235; 0.606)	0.177 (0.112; 0.279)
Main Islands	0.678 (0.361; 1.274)	0.434 (0.230; 0.818)	0.135 (0.065; 0.280)
Any underlying risk factor	0.379 (0.211; 0.679)	3.464 (1.757; 6.828)	1.752 (0.960; 3.198)
Any need for respiratory support	-		
NICU admission	-		
Immunization with nirsevimab	0.130 (0.090; 0.190)	0.350 (0.248; 0.493)	0.441 (0.233–0.837)
RSV-positive status	-	-	-

**Figure A3:**
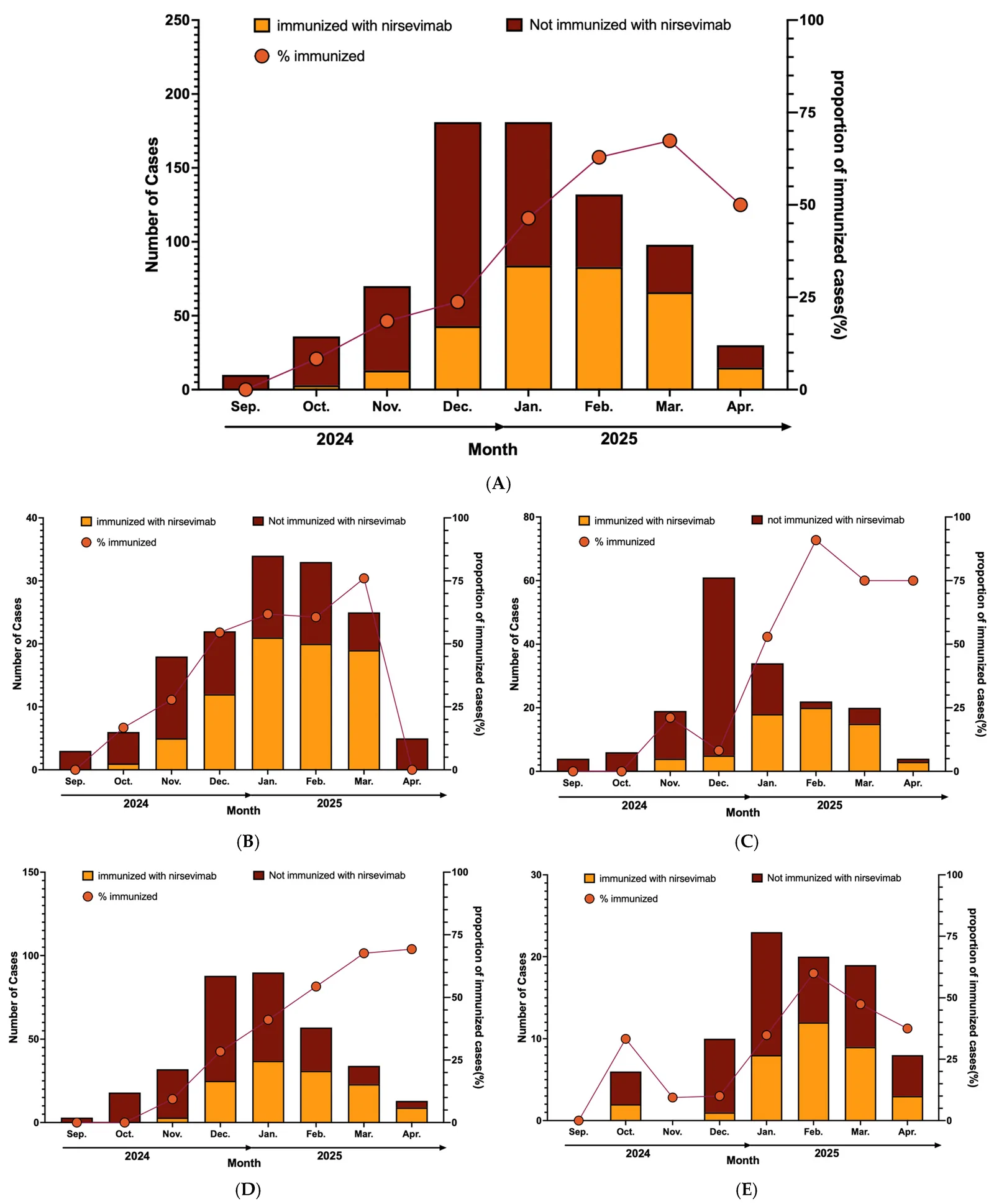
Monthly proportion of infants hospitalized for lower respiratory tract infections who had previously received nirsevimab immunization during the 2024–2025 surveillance period. Panel (**A**) shows the overall monthly proportion, whereas panels (**B**–**E**) report the corresponding trends for Northern Italy, Central Italy, Southern Italy, and the Major Islands, respectively. Monthly proportions were calculated as the number of nirsevimab-immunized infants divided by the total number of LRTI hospitalizations recorded in the corresponding month and geographical area.

## Data Availability

Data are available from the corresponding authors and from the Italian Society of Neonatology.

## Supplementary Materials

The following supporting information can be downloaded at: https://www.mdpi.com/article/10.3390/v18090982/s1, Supplementary File S1: STROBE Statement—Checklist of items that should be included in reports of case-control studies.

## Abbreviations

The following abbreviations are used in this manuscript:

RSV	Respiratory Syncytial Virus
NICU	Neonatal Intensive Care Unit
CPAP	Continuous Positive Airway Pressure
aOR	Adjusted Odds Ratio
CI	Confidence Intervals
LRTI	Lower respiratory tract infection
SD	Standard Deviation
TMLE	Targeted Maximum Likelihood Estimation
GA	Gestational Age
RS	Respiratory Support

## Appendix A

**Table A1:** Centers participating in the Italian Working Group on RSV prophylaxis of Italian Society of Neonatology. Potential catchment population was calculated based on the pediatric population aged <2 years and <1 year in the parent province (Second Level administrative division, NUTS 3) from each participating center.

Italian Region	No. of Centers	Name of the Center (in Italian)	Province	Potential Catchment Population (No.)	
				<2 Years of Age (/Total, %)	<1 Year of Age (/Total, %)
Northern Italy					
Autonomous Province of Bozen	1	TIN Ospedale di Bolzano	Bolzano	9245 (1.86%)	4674 (1.91%)
Autonomous Province of Trento	1	TIN Ospedale di Trento	Trento	7518 (1.51%)	3736 (1.53%)
Emilia Romagna	5	TIN AOU di Modena	Modena	9604 (1.93%)	4769 (1.95%)
		Neonatologia Ospedale di Parma	Parma	6158 (1.24%)	3078 (1.26%)
		Neonatologia Ospedale di Reggio Emilia	Reggio nell’Emilia	7010 (1.41%)	3575 (1.46%)
		TIN e Neonatologia Ospedale di Rimini	Rimini	3698 (0.74%)	1760 (0.72%)
		TIN AOU Ferrara	Ferrara	3515 (0.71%)	1718 (0.71%
Friuli Venezia Giulia	1	Neonatologia Azienda Sanitaria Universitaria Friuli Centrale di Udine	Udine	5533 (1.11%)	2656 (1.09%)
Lombardy	13	Patologia Neonatale Ospedale di Magenta	Milan	42,904 (8.61%)	21,444 (8.77%)
		TIN PO Macedonio Melloni di Milano			
		Patologia Neonatale Ospedale San Raffaele di Milano			
		SC Neonatologia e Terapia Intensiva Neonatale Fondazione IRCCS Ca’ Granda Ospedale Maggiore Policlinico di Milano			
		Neonatologia e TIN Ospedale S. Matteo di Pavia	Pavia	6741 (1.35%)	3325 (1.36%)
		Patologia Neonatale ASST Papa Giovanni XXIII di Bergamo	Bergamo	14,646 (2.94%)	7214 (2.95%)
		TIN Ospedale di Seriate			
		TIN Ospedale S. Gerardo dei Tintori di Monza	Monza-Brianza	11,200 (2.25%)	5492 (2.25%)
		TIN Fondazione Poliambulanza di Brescia	Brescia	16,605 (3.33%)	8197 (3.35%)
		TIN Spedali Civili di Brescia			
		TIN Ospedale di Lecco	Lecco	4088 (0.82%)	1955 (0.80%)
		TIN Ospedale di Mantova	Mantua	5004 (1.00%)	2461 (1.01%)
		TIN e Neonatologia Ospedale di Varese	Varese	10,956 (2.20%)	5386 (2.20%)
Piedmont	8	TIN Ospedale Sant’Anna Torino Clinica Universitaria	Turin	25,311 (5.08%)	12,350 (5.05%)
		TIN Ospedale Sant’Anna Torino Clinica Ospedaliera			
		TIN- Neonatologia Ospedale Santa Croce di Moncalieri			
		TIN Ospedale Maria Vittoria di Torino			
		SC Terapia Intensiva Neonatale e Neonatologia ASO S. Croce e Carle di Cuneo	Cuneo	7675 (1.54%)	3796 (1.55%)
		TIN AOU SS Antonio e Biagio e C. Arrigo di Alessandria	Alessandria	4201 (0.84%)	2134 (0.87%)
		TIN Ospedale Maggiore della Carità di Novara	Novara	4463 (0.90%)	2215 (0.91%)
		Patologia Neonatale Ospedale di Ponderano-Biella	Biella	1622 (0.33%)	809 (0.33%)
Central Italy					
Latium	12	Neonatologia e Pediatria Ospedale di Formia	Latina	6828 (1.37%)	3371 (1.38%)
		Neonatologia e Pediatria Ospedale di Latina			
		Neonatologia-Patologia Neonatale-TIN-Policlinico Casilino di Roma	Rome	49,643 (9.97%)	24,026 (9.83%)
		Neonatologia e TIN AO San Camillo di Roma			
		Patologia Neonatale, Ospedale Sandro Pertini di Roma			
		Terapia Semi-Intensiva Neonatale, Ospedale Pediatrico Bambino Gesù di Roma			
		TIN Policlinico A. Gemelli di Roma			
		TIN Policlinico Umberto I di Roma			
		Neonatologia e TIN Ospedale S. Eugenio di Roma			
		Neonatologia e Pediatria Ospedale dei Castellli di Roma			
		Neonatologia e TIN AO San Giovanni Addolorata di Roma			
		Pediatria Ospedale di Civitavecchia			
Marche	2	Terapia Intensiva Neonatale Ospedale Pediatrico G. Salesi di Ancona	Ancona	5193 (1.04%)	2633 (1.08%)
		Neonatologia Ospedale di Macerata	Macerata	3557 (0.71%)	1753 (0.72%)
Tuscany	2	Neonatologia e TIN AOU Pisana	Pisa	5083 (1.02%)	2499 (1.02%)
		TIN Azienda Ospedaliero-Universitaria di Siena	Siena	3041 (0.61%)	1479 (0.61%)
Umbria	2	TIN Azienda Ospedaliera di Perugia	Perugia	7059 (1.42%)	3409 (1.39%)
		Patologia Neonatale Azienda Ospedaliera di Terni	Terni	2219 (0.45%)	1089 (0.45%)
Southern Italy					
Abruzzo	3	Neonatologia Ospedale di Chieti	Chieti	4129 (0.83%)	2005 (0.82%)
		Neonatologia e TIN Ospedale de L’Aquila	L’Aquila	3268 (0.66%)	1549 (0.63%)
		Neonatologia Ospedale di Pescara	Pescara	3601 (0.72%)	1763 (0.72%)
Apulia	5	TIN Ospedale Regionale F. Miulli di Acquaviva delle Fonti	Bari	15,385 (3.09%)	7433 (3.04%)
		AUOC Policlinico di Bari			
		TIN Ospedale Di Venere di Bari			
		Ospedale SS Annunziata di Taranto	Taranto	6268 (1.26%)	3050 (1.25%)
		TIN AO Cardinale G. Panico di Tricase, Lecce	Lecce	8813 (1.77%)	4313 (1.76%)
Basilicata	1	TIN Ospedale San Carlo di Potenza	Potenza	3731 (0.75%)	1825 (0.75%)
Calabria	3	Patologia Neonatale Ospedale di Catanzaro	Catanzaro	4352 (0.87%)	2100 (0.86%)
		Neonatologia Ospedale Annunziata di Cosenza	Cosenza	8326 (1.67%)	4074 (1.67%)
		Neonatologia GOM di Reggio Calabria	Reggio di Calabria	7439 (1.49%)	3613 (1.48%)
Campania	14	TIN Ospedale Villa dei Fiori di Acerra	Naples	44,787 (8.99%)	21,925 (8.97%)
		TIN Ospedale di Castellammare			
		TIN AORN Cardarelli di Napoli			
		TIN AOU Monaldi di Napoli			
		TIN Ospedale Santobono di Napoli			
		TIN Ospedale Evangelico Betania di Napoli			
		TIN Ospedale Fatebenefratelli di Napoli			
		TIN Università Federico II di Napoli			
		TIN Università L. Vanvitelli			
		TIN Ospedale di Avellino	Avellino	4820 (0.97%)	2366 (0.97%)
		TIN Ospedale San Pio di Benevento	Benevento	3246 (0.65%)	1526 (0.62%)
		TIN Ospedale di Caserta	Caserta	13,894 (2.79%)	6829 (2.79%)
		TIN Ospedale di Salerno	Salerno	14,315 (2.87%)	7010 (2.87%)
		TIN Ospedale di Nocera			
Sardinia	1	Patologia Neonatale AOU Monserrato di Cagliari	Cagliari	3378 (0.68%)	1581 (0.65%)
Sicily	13	TIN e Neonatologia Ospedale di Agrigento	Agrigento	5743 (1.15%)	2750 (1.13%)
		TIN AOE Cannizzaro di Catania	Catania	15,797 (3.17%)	7683 (3.14%)
		TIN Ospedale Garibaldi di Catania			
		TIN Ospedale G. Rodolico di Catania			
		Neonatologia Policlinico Umberto I di Enna	Enna	1852 (0.37%)	917 (0.38%)
		Neonatologia AOUP “Paolo Giaccone” di Palermo	Palermo	17,219 (3.46%)	8448 (3.46%)
		TIN Ospedale Buccheri La Ferla di Palermo			
		UTIN e Neonatologia Ospedale di Siracusa	Siracusa	4870 (0.98%)	2424 (0.99%)
		Neonatologia Ospedale di Trapani	Trapani	5500 (1.10%)	2760 (1.13%)
		Neonatologia Ospedale di Taormina	Messina	7084 (1.42%)	3488 (1.43%)
		Patologia Neonatale e TIN AOU G. Martino di Messina			
		Pediatria e TIN P.O. Patti Barone Romeo–Messina di Patti			
		Pediatria Ospedale di Milazzo			
Total Potential catchment population	88	-	-	498,137 (100%)	244,435 (100%)

**Table A2:** Content of the SIN–SIP Survey on hospital admissions for bronchiolitis during the 2024–2025 epidemic season (Author’s translation).

Item	Please Enter Data for Patients Admitted with Bronchiolitis of Any Etiology, Whether Known or Unknown, Between 1 September 2024 and 30 April 2025.
1. Discharging unit	(please report complete name) _________________________________________________
1B. Region	Piedmont Aosta Valley Lombardy Trentino–South Tyrol Veneto Friuli Venezia Giulia Liguria Emilia-Romagna Tuscany Umbria Marche Lazio Abruzzo Molise Campania Apulia Basilicata Calabria Sicily Sardinia
1C	City (please report) _________________________________________________________
2	Code of the discharging unit (please choose one code) 31. Neonatology 62. Neonatal Care Unit 73. Neonatal Intensive Care Unit–NICU
3	Patient number: ___________________________________________
4	Date of birth (DD/MM/YYYY): ______________________________
5	Positive RSV test: ( ) yes/( ) no/( ) unknown status/not performed
6	Date of admission (DD/MM/YYYY): ______________________________
7	Date of discharge (DD/MM/YYYY): ______________________________
8	Admission to a Neonatal Intensive Care Unit: ( ) yes/( ) no/( ) unknown status
9	Respiratory support (multiple answers may be selected) ( ) None ( ) Low-flow oxygen ( ) High-flow oxygen ( ) CPAP or another form of non-invasive ventilation ( ) Invasive mechanical ventilation ( ) Other
10	Nirsevimab prophylaxis ( ) yes ( ) no ( ) no, due to maternal RSV vaccination ( ) unknown status
11	Palivizumab prophylaxis initiated ( ) yes ( ) no ( ) unknown status
12	Risk factors (please choose one option only) ( ) none ( ) prematurity ( ) congenital heart disease ( ) chronic lung disease ( ) neuromuscular disease ( ) Immunosuppression
13	Final outcome at hospital discharge ( ) discharged home ( ) transferred to another hospital ( ) died ( ) other: ________________________________

**Table A3:** Estimates on immunization effectiveness of Nirsevimab (mAb) against lower respiratory tract infection (LRTI) by respiratory syncytial virus (RSV).

Condition		RSV-Associated LRTI		Non-RSV-Associated LRTI		IE	95% CI
		mAb yes (N)	mAb no (N)	mAb yes (*n*)	mAb no (*n*)		
Hospitalization							
By area							
Northern Italy	*n*/146, %	40 (27.40%)	44 (30.14%)	38 (26.03%)	24 (16.44%)	42.6%	−11.8; 70.5
Central Italy	*n*/170, %	21 (12.35%)	71 (41.76%)	44 (25.88%)	34 (20.00%)	77.1%	55.7; 88.2
Southern Italy	*n*/335, %	34 (10.15%)	139 (41.49%)	94 (28.06%)	68 (20.30%)	81.3%	71.2; 89.1
Islands	*n*/86, %	9 (10.47%)	40 (46.51%)	26 (30.23%)	11 (12.79%)	90.5%	74.9; 96.5
By gestational age							
Preterm	*n*/55, %	7 (12.73%)	15 (27.27%)	24 (43.64%)	91 (16.36%)	82.5%	43.1; 94.6
Full-term	*n*/682, %	97 (14.22%)	279 (40.91%)	178 (26.10%)	128 (18.77%)	75.0%	65.4; 81.9
By age at admission							
<7 days	*n*/34, %	6 (12.73%)	11 (27.27%)	6 (17.65%)	11 (32.35%)	-	-
7 to 29 days	*n*/500, %	59 (11.80%)	193 (32.35%)	138 (27.60%)	110 (22.0%)	75.6%	64.2; 83.4
30 to 59 days	*n*/130, %	27 (20.77%)	60 (46.15%)	36 (27.69%)	7 (5.38%)	91.2%	77.9; 96.5
60 to 89 days	*n*/42, %	8 (19.05%)	16 (38.10%)	11 (26.19%)	7 (16.67%)	68.2%	−13.5; 91.1
≥3 months	*n*/16%	2 (12.50%)	5 (31.25%)	2 (12.50%)	7 (43.75%)	88.6%	−10.8; 98.8
By comorbidities							
None	*n*/655, %	94 (14.35%)	275 (41.98%)	166 (25.34%)	120 (18.32%)	75.3%	65.6; 82.3
Any	*n*/82, %	10 (12.20%)	19 (23.17%)	36 (43.90%)	17 (20.73%)	75.1%	35.2; 90.5
Total (crude)	*n*/737, %	104 (14.115)	294 (38.89%)	202 (27.41%)	137 (18.59%)	76.0%	67.2; 82.4
Total (adjusted) *						78.3%	69.6; 84.5
Need for respiratory support							
Total (crude)	*n*/471, %	72 (15.29%)	238 (50.53%)	97 (20.59%)	64 (13.59%)	80.0%	69.9; 86.8
Total (multivariable) *						80.2%	68.1; 87.7
NICU admission							
Total (crude)	*n*/145, %	32 (22.07%)	58 (40.00%)	31 (21.38%)	24 (16.55%)	57.3%	15.2; 78.5
Total (multivariable) *						64.3%	16.7; 73.3

***** Adjusted estimates were calculated by means of targeted maximum likelihood estimation, including in the model age at admission, prematurity, month of hospitalization, and geographical region.

**Table A4:** Main outcome variables of Neonatal Intensive Care Unit (NICU) admission, need for respirator support, in general, and for high-flow O2 therapy and/or CPAP and/or invasive respiratory support by main geographic areas. Risk Ratios (RRs) and their respective 95% confidence intervals (95% CI) were calculated by assuming Northern Italy as the reference group.

	Northern Italy (N./146, %)	Central Italy (N./170, %)	Southern Italy (N./335, %)	Main Islands (N./86, %)
NICU admission	72, 49.3% (REFERENCE)	13, 7.6% RR 0.155 (95% CI 0.090; 0.268)	49, 14.6% RR 0.297 (95% CI 0.219; 0.403)	11, 12.8% RR 0.259 (95% CI 0.146; 0.461)
Respiratory support (any)	114, 78.1% (REFERENCE)	99, 58.2% RR 0.631 (95% CI 0.535; 0.744)	201, 60.0% RR 0.768 (95% CI 0.680; 0.869)	57, 66.3% RR 0.849 (95% CI 0.714; 1.010)
High-flow O2 therapy	87, 59.6% (REFERENCE)	78, 45.9% RR 0.770 (95% CI 0.624; 0.951)	129, 38.5% RR 0.646 (95% CI 0.534; 0.782)	42, 48.8% RR 0.820 (95% CI 0.634; 1.057)
CPAP	43, 29.5% (REFERENCE)	36, 21.2% RR 0.719 (95% CI 0.490; 1.055)	99, 29.6% RR 1.003 (95% CI 0.743; 1.355)	26, 30.2% RR 1.027 (95% CI 0.689; 1.543)
Invasive respiratory support	5, 3.4% (REFERENCE)	0, - RR 0.086 (95% CI 0.005; 1.559)	1, 0.3% RR 0.088 (95% CI 0.010; 0.739)	1, 1.2% RR 0.340 (95% CI 0.040; 2.858)

**Table A5:** Summary of recall strategies for infants born outside the epidemic season [88], and proportion of children < 1 year of age during the respiratory syncytial virus seasonal epidemic of 2024–2025 (https://demo.istat.it; data retrieved on 25 April 2026).

Targeted Children	Regions	Children < 1 Year of Age During the RSV Season 2024–2025 (No./358,173; %)
Born after January 2024	Piedmont	24,122 (6.73%)
	Lombardy	63,745 (17.80%)
	Veneto	29,169 (8.14%)
	Sicily	32,328 (9.03%)
Born after April 2024	Aosta Valley	684 (0.19%)
	Autonomous Province of Trento	3736 (1.04%)
	Autonomous Province of Bolzano	4674 (1.30%)
	Friuli Venezia Giulia,	6704 (1.87%)
	Tuscany	20,138 (5.62%)
Born after July 2024	Liguria	8143 (2.27%)
	Apulia	22,899 (6.39%)
	Calabria	11,936 (3.33%)
Born after August 2024	Latium	32,099 (8.96%)
	Campania	39,656 (11.07%)
Born between November 2024 and March 2025	Umbria	4498 (1.26%)
	Marche	8293 (2.32%)
	Abruzzo	7016 (1.96%)
	Basilicata	2968 (0.83%)
	Sardinia	6486 (1.81%)
Other/No catch-up campaign	Emilia Romagna	27,287 (7.62%)
	Molise	1502 (0.42%)

**Table A6:** Pathogens associated with hospital admissions due to lower respiratory tract infections.

Pathogen	Total Positive Specimens (N/737, %)	Coinfections with RSV (*n*/N, %)
Human Metapneumovirus	10, 1.36%	0, -
Adenovirus	0, -	0, -
Rhino-, Enterovirus	40, 5.43%	6, 15.00%
Parainfluenza virus	6, 0.81%	1, 16.67%
Seasonal influenza virus	10, 1.36%	1, 10.00%
Non-SARS-CoV-2 Coronaviruses	9, 1.22%	2, 22.22%
Bacterial infections	6, 0.81%	5, 83.33%
*Haemophilus influenzae*	4, 0.54%	3, 75.00%
*Streptococcus pneumoniae*	1, 0.14%	1, 100%
*Staphylococcus aureus*	1, 0.14%	1, 100%

## Appendix B

Full list of the members of Italian Working Group on RSV Prophylaxis of Italian Society of Neonatology:

**Family Name,** **Personal Name**	**Title**	**Province**	**City**	**Name of the Center**	
AGOSTI Massimo	PROF.	Varese	Varese	Hospital of Varese—University of Insubria	NICU and Neonatology
ALFANO Carmela	DR.	Avellino	Avellino	Hospital of Avellino	NICU
ALFANO Sara	DR.	Naples	Castellammare di Stabia	Hospital of Castellammare	NICU
ANCORA Gina	DR.	Rimini	Rimini	Rimini Hospital	NICU and Neonatology
BALDASSARRE Mariella	PROF.	Bari	Bari	AOUC Policlinico di Bari	NICU
BARERA Graziano	DR.	Milan	Milan	“San Raffaele” Hospital	Neonatal Pathology
BELLAN Cristina	DR.	Bergamo	Seriate	Hospital of Seriate	NICU
BERARDI Alberto	PROF.	Modena	Modena	Azienda Ospedaliero Universitaria di Modena	NICU
BETTA Mary	DR	Catania	Catania	“G Rodolico” Hospital	NICU-Neonatology
BETTO Ada	DR.	MESSINA	Milazzo	“V.Fogliani” Hospital	Neonatology and Pediatrics
BONANNO Elvira	DR.	Cosenza	Cosenza	“Annunziata” Hospital	Neonatology Unit
BOTTIGLIERI Carlotta	DR.	Rome	Rome	“Sandro Pertini” Hospital	Neonatal Pathology Unit
BURATTINI Ilaria	DR.	Ancona	Ancona	“G. Salesi Pediatric Hospital”	NICU
CACACE Caterina	DR.	Messina	Patti	“Barone Romeo” Hospital	NICU and Pediatrics
CANDELA Gilberto	DR.	Reggio di Calabria	Reggio di Calabria	Great Metropolitan Hospital	Neonatology Unit
CAPOZZI Laura	DR.	Naples	Naples	“Monaldi” Hospital	NICU
CAPRIO Angela Maria	DR.	Caserta	Caserta	Hospital of Caserta	NICU
CARBONARA Caterina	DR.	Turin	Turin	“Sant’Anna” Hospital	NICU and Neonatology
CELI Federica	DR.	Terni	Terni	Azienda Ospedaliera di Terni	NICU and Neonatal Pathology Unit
CONDO’ Manuela	DR.	Lecco	Lecco	ASST Lecco	NICU
COSCIA Alessandra	DR.	Turin	Turin	“Sant’Anna” Hospital—University of Turin	NICU and Neonatology Unit
COSTA Simonetta	DR.	Rome	Rome	“Policlinico Casilino” Hospital	Neonatology and Neonatal Pathology Unit
CURTOPELLE Maria	DR.	Agrigento	Agrigento	“San Giovanni di Dio” Hospital	NICU and Neonatology
D’AMICO Pietro	DR.	Catania	Catania	“Cannizzaro” Hospital	NICU and Neonatology
DE BERNARDO Giuseppe	DR.	Naples	Naples	“Fatebenefratellli” Hospital	NICU
DE COSMO Lucrezia	DR.	Taranto	Taranto	“SS Annunziata” Hospital	NICU
DI FABIO Sandra	DR.	L’Aquila	L’Aquila	Hospital of L’Aquila	NICU and Neonatology
DI MANSO Gaetano	DR.	Benevento	Benevento	“San Pio” Hospital	NICU
DI VALERIO Susanna	DR.	Pescara	Pescara	Hospital of Pescara	NICU and Neonatology
DICHIO Silvia	DR.	MIlan	Milan	“Macedonio Melloni” Hospital	NICU
DITURI Francesco	DR.	Rome	Civitavecchia	Hospital of Civitavecchia	Pediatric Unit
DOTTA Andrea	DR.	Rome	Rome	Pediatric Hospital “Bambino Gesù”	NICU and neonatal sub-intensive care unit
FARRUGGIA Pietro	DR.	Palermo	Termini Imerese	“S Cimino” Hospital	Pediatrics
FASOLATO Valeria	DR.	Mantua	Mantua	“Carlo Poma” Hospital	NICU
FERRAZZOLI Federica	DR.	Rome	Rome	“Sant’Eugenio” Hospital	NICU and Neonatology Unit
FIOCCHI Stefano	DR.	MIlan	Magenta	Hospital of Magenta	Neonatal Pathology Unit
FRASCOGNA Anna Rita	DR.	Salerno	Salerno	Hospital of Salerno	NICU
FRATELLANZA Annagrazia	DR.	Naples	Naples	Azienda Ospedaliera di Rilievo Nazionale CARDARELLI	NICU
FUMAGALLI Monica	DR.	MIlan	MIlan	“Fondazione IRCCS Ca’ Granda, Ospedale Maggiore Policlinico”	NICU and neonatology
GARGANO Giancarlo	DR.	Reggio nell’Emilia	Reggio nell’Emilia	Azienda USL—IRCCS di Reggio Emilia	Neonatology
GAZZOLO Diego	DR.	Chieti	Chieti	Hospital of Chieti	Neonatology
GHIRARDELLO Stefano	DR	Pavia	Pavia	“San Matteo” Hospital	NICU and Neonatology
GITTO Eloisa	PROF.	Messina	Messina	“G Martino Hospital”	NICU and Neonatal Pathology
GIUFFRE’ Mario	PROF.	Palermo	Palermo	“P Giaccone” Hospital	NICU and Neonatology
GUCCIONE Paolo	DR.	Messina	Taormina	Hospital of Taormina	NICU
LA PLACA Simona	DR.	Trapani	Trapani	“San Antonio Abate” Hospital	NICU and Neonatology
LAGO Paola	DR.	Treviso	Treviso	Hospital of Treviso	NICU
LATORRE Giuseppe	DR.	Bari	Acquaviva delle Fonti	“F Miulli” Regional Hospital	NICU
LORENZONI Patrizia	DR.	Pisa	Pisa	Azienda Ospedaliero Universitaria Pisana	NICU and Neonatology
LUBRANO Riccardo	DR.	Latina	Latina	Hospital of Latina	Neonatology and Pediatrics
MAFFEI Gianfranco	DR.	Foggia	Foggia	“Ospedali Riuniti di Foggia”	NICU and Neonatology
MAGGIO Luca	DR.	Rome	Rome	“San Camillo” Hospital	NICU and Neonatology Unit
MANZONI Paolo	PROF.	Biella	Ponderano	Ponderano Hospital	Neonatal Pathology Unit
MARAGLIANO Giovanna	DR.	Rome	Rome	“Castelli di Roma” Hospital	Neonatology and Pediatrics
MAROZZI Giulia	DR.	Macerata	Macerata	Hospital of Macerata	Neonatology
MARZANO MAria Grazia	DR.	Rome	Formia	Hospital of Formia	Neonatology and Pediatrics
MORETTI Sabrina	DR.	Parma	Parma	Azienda Ospedaliero Universitaria di Parma	Neonatology
MORREALE Sabrina	DR.	Enna	Enna	“Umberto I” Hospital	NICU and neonatology
NUCCIO Melissa	DR	Lecce	Tricase	“Cardinale G Panico” Hospital	NICU
ONORATI Lorenza Rosangela	DR.	Bolzano	Bolzano	Hospital of Bolzano	NICU
OTTONELLO Giovanni	DR.	Cagliari	Cagliari	“Monserrato” Hospital	Neonatal Pathology Unit
PALATTA Sara	DR.	Rome	Rome	San Giovanni Addolorata Hospital	NICU and Neonatology Unit
PALOMBO Daniele	DR.	Siena	Siena	Azienda Ospedaliero Universitaria di Siena	NICU
PATERLINI Giuseppe	DR.	Brescia	Brescia	“Fondazione Poliambulanza” Hospital	NICU
PERNICIARO Simona	DR	Varese	Varese	Hospital of Varese—University of Insubria	NICU and Neonatology
PIERRI Luca	DR.	Naples	Naples	“Santobono” Hospital	NICU
PIROZZI Emilia	DR.	Naples	Naples	“Villa dei Fiori” Hospital	NICU
PITTINI Carla	DR.	Udine	Udine	Azienda SAnitaria Universitaria Friuli Centrale	Neonatology
QUERCIA Michele	DR.	Bari	Bari	“Di Venere” Hospital	NICU
RICOTTI Alberto	DR.	Alessandria	Alessandria	“Azienda Ospedaliero Universitaria SS Antonio e Biagio e C Arrigo”	NICU and Neonatology
RISSO Francesco Maria	DR.	Brescia	Brescia	“Spedali Civili” Hospital	NICU
ROSETO Vincenza	DR.	Naples	Naples	“Ospedale Evangelico Betania”	NICU
SANNIA Andrea	DR.	Cuneo	Cuneo	“ASO S Croce e Carle” Hospital	NICU and Neonatology Unit
SANVANT LEVET Patrizia	DR.	Turin	Turin	“Maria Vittoria” Hospital	NICU and Neonatology
SAPORITO Alessandro	DR.	Catania	Catania	“San Marco” Hospital	NICU and Neonatology
SCAVONE Maria	DR.	Potenza	Potenza	“San Carlo” Hospital	NICU
SCHIAVO Claudia	DR.	Salerno	Nocera Inferiore	Hospital of Nocera Inferiore	NICU
SOLINAS Agostina	DR.	Ferrara	Ferrara	Azienda Ospedaliero Universitaria di Ferrara	NICU
SPAGNOLO Simona	DR.	Catanzaro	Catanzaro	Hospital of Catanzaro	Neonatal Pathology Unit
SPAGNUOLO Ferdinando	DR.	Naples	Naples	“Luigi Vanvitelli” University	NICU
TERMINI Donatella	DR.	Palermo	Palermo	“Buccheri La Ferla” Hospital	NICU and Neonatology
TINA Gabriella	DR.	Catania	Catania	“ARNAS Garibaldi” Hospital	NICU and Neonatology
TIRANTELLO Massimo	DR.	Siracusa	Siracusa	“Umberto I” Hospital	NICU and Neonatology
TOTA Francesca	DR.	Trento	Trento	Hospital of Trento	NICU
TROIANI Stefania	DR.	Perugia	Perugia	Azienda Ospedaliera di Perugia	NICU
TZIALLA Chryssoula	DR.	Bergamo	Bergamo	Papa Giovanni XXIII Hospital	Neonatal Pathology
UMBALDO Angela	DR.	Naples	Naples	“Federico II” University	NICU and Neonatology
VENTO Giovanni	DR.	Rome	Rome	“Policlinico Agostino Gemelli” Hospital	NICU
VENTURA Maria Luisa	DR.	Monza Brianza	Monza	“San Gerardo dei Tintori” Hospital	NICU
VIVALDA Mauro	DR.	Turin	Moncalieri	“Santa Croce” Hospital	NICU and Neonatology
