# Supplementary material for: Real-World Effectiveness of Nirsevimab Against RSV-LRTI and Bronchiolitis Hospitalization: Results from the 2024–2025 Italian Universal Immunization Campaign in Newborns
Source: Viruses. 2026 Sep 7;18(9):982. doi: 10.3390/v18090982 (PMC13611971; doi:10.3390/v18090982)
Supplement: Supplementary file 1 [file viruses-18-00982-s001.zip › viruses-4488153-supplementary.pdf]

STROBE Statement—Checklist of items that should be included in reports of case-control studies

Manuscript: Real-World Effectiveness of Nirsevimab against RSV-LRTI and Bronchiolitis Hospitalization: Results from the 2024–2025 Italian Universal Immunization Campaign in Newborns

Checklist prepared from the revised manuscript supplied by the authors. Page references refer to the current manuscript pagination.

Section	Item	STROBE recommendation	Reported on page(s) / manuscript location
**Title and abstract**	1(a)	Indicate the study's design with a commonly used term in the title or the abstract.	pp. 1–3 (title; Abstract: multicenter retrospective test-negative study)
	1(b)	Provide in the abstract an informative and balanced summary of what was done and what was found.	pp. 2–3 (Abstract)
**Introduction**	2	Explain the scientific background and rationale for the investigation being reported.	pp. 3–4 (Introduction)
	3	State specific objectives, including any prespecified hypotheses.	p. 4 (end of Introduction: study aim)
**Methods**	4	Present key elements of study design early in the paper.	p. 5, Section 2.1 (multicenter retrospective observational study; test-negative design)
	5	Describe the setting, locations, and relevant dates, including periods of recruitment, exposure, follow-up, and data collection.	pp. 5–6, Section 2.1; Appendix Table A1 (88 Italian centers; 1 Sep 2024–30 Apr 2025; regional implementation timing)
	6(a)	Give eligibility criteria, and the sources and methods of case ascertainment and control selection. Give the rationale for the choice of cases and controls.	pp. 5–6, Sections 2.1–2.2 (inclusion/exclusion criteria; RSV-positive cases and RSV-negative hospitalized LRTI controls)
	6(b)	For matched studies, give matching criteria and the number of controls per case.	N/A — the study was not individually matched.
	7	Clearly define all outcomes, exposures, predictors, potential confounders, and effect modifiers. Give diagnostic criteria, if applicable.	pp. 5–8, Sections 2.1–2.3 (LRTI definition; nirsevimab exposure; RSV status; secondary outcomes; covariates)
	8	For each variable of interest, give sources of data and details of methods of assessment. Describe comparability of assessment methods if there is more than one group.	pp. 5–6, Sections 2.1–2.2 (hospital discharge records; structured questionnaire; immunization records/documentation; RT-PCR or antigen testing)
	9	Describe any efforts to address potential sources of bias.	pp. 6–8 (consecutive inclusion, duplicate/inter-hospital transfer check, standardized definitions/data collection, multivariable adjustment/TMLE); pp. 23–25 (limitations)
	10	Explain how the study size was arrived at.	pp. 5–6 and p. 8; all eligible consecutive admissions during the predefined surveillance period were included; no formal a priori sample-size calculation reported.
	11	Explain how quantitative variables were handled in the analyses. If applicable, describe which groupings were chosen and why.	pp. 6–8, Section 2.3 (continuous-variable distribution testing; age strata; month/geographic groupings; descriptive and regression handling)
	12(a)	Describe all statistical methods, including those used to control for confounding.	pp. 6–8, Section 2.3 (univariate tests, logistic regression, crude IE, TMLE/Super Learner adjustment)
	12(b)	Describe any methods used to examine subgroups and interactions.	pp. 7–8, Section 2.3; Results Section 3.4 (age-stratified and other subgroup IE analyses; exploratory age analysis among immunized infants)
	12(c)	Explain how missing data were addressed.	p. 7, Section 2.3 (listwise deletion for analysis-specific missingness)
	12(d)	If applicable, explain how matching of cases and controls was addressed.	N/A — no individual matching was performed.
	12(e)	Describe any sensitivity analyses.	No formal sensitivity analysis is reported;

			exploratory subgroup and outcome-specific analyses are described separately.
**Results**	13(a)	Report numbers of individuals at each stage of the study (potentially eligible, examined for eligibility, confirmed eligible, included, analysed).	pp. 8–9, Section 3.1 and Figure 1 (2,835 admissions; 768 aged ≤24 months; 737 included/analysed)
	13(b)	Give reasons for non-participation at each stage.	p. 9, Figure 1 (age exclusion and incomplete personal data reporting)
	13(c)	Consider use of a flow diagram.	p. 9, Figure 1
	14(a)	Give characteristics of study participants and information on exposures and potential confounders.	pp. 11, 16–17; Tables 2–3 (demographic, geographic, comorbidity, immunization, RSV and clinical characteristics)
	14(b)	Indicate number of participants with missing data for each variable of interest.	Missingness handling is described in Methods; incomplete records are addressed in the flow diagram. Variable-specific missing counts are not separately tabulated where data are complete for the analytical variables.
	14(c)	Summarize follow-up time (e.g., average and total amount).	N/A — retrospective hospitalization-based test-negative study without longitudinal follow-up.
	15	Report numbers in each exposure category, or summary measures of exposure.	pp. 8, 11, 16–17; 306 nirsevimab-immunized and 431 non-immunized infants; Tables 2–3.
	16(a)	Give unadjusted estimates and, if applicable, confounder-adjusted estimates and their precision. Make clear which confounders were adjusted for and why they were included.	pp. 14–19, Sections 3.2–3.4; Tables 3–5 and Appendix Table A3 (crude estimates, aORs, TMLE estimates with 95% CIs; adjustment set described in Methods)
	16(b)	Report category boundaries when continuous variables were categorized.	pp. 7, 9–10, 15 and Section 3.4 (age categories; calendar-period groupings)
	16(c)	If relevant, consider translating estimates of relative risk into absolute risk for a meaningful time period.	Not applicable to the primary test-negative estimand; TMLE marginal probabilities of RSV positivity under immunization and no immunization are reported in Section 3.4.
	17	Report other analyses done—e.g., analyses of subgroups and interactions, and sensitivity analyses.	pp. 12–19, Sections 3.2–3.4 and Appendix (monthly/geographic analyses, age-stratified analyses, subgroup crude IE, secondary outcome analyses).
**Discussion**	18	Summarize key results with reference to study objectives.	pp. 19–20, Section 4.1
	19	Discuss limitations of the study, taking into account sources of potential bias or imprecision. Discuss both direction and magnitude of potential bias.	pp. 23–25, Section 4.3.1 (residual confounding, selection/generalizability, regional implementation, multiplicity, TMLE learner diversity/positivity, pathogen data, coverage data, unmeasured factors, follow-up/costs)
	20	Give a cautious overall interpretation of results considering objectives, limitations, multiplicity of analyses, results from similar studies, and other relevant evidence.	pp. 20–26, Sections 4.2–4.4 and Conclusions
	21	Discuss the generalisability (external validity) of the study results.	pp. 20–25, Sections 4.2–4.3 (multicenter/multiregional representation; hospitalized neonatal-setting population; limitations to broader generalization)
**Other information**	22	Give the source of funding and the role of the funders for the present study and, if applicable, for the original study on which the present article is based.	p. 26 (Funding: no external funding; publication fees to be covered by the Italian Society of Neonatology)

Note: This checklist maps the current revised manuscript to the STROBE case-control checklist. “N/A” indicates an item not applicable to this unmatched, hospitalization-based test-negative study. Page numbers should be rechecked if journal typesetting changes pagination.
